# Guidance for Canadian Breast Cancer Practice: National Consensus Recommendations for the Systemic Treatment of Patients with HER2+ Breast Cancer in Both the Early and Metastatic Setting

**DOI:** 10.3390/curroncol31110484

**Published:** 2024-10-24

**Authors:** Mita Manna, Karen A. Gelmon, Jean-François Boileau, Christine Brezden-Masley, Jeffrey Q. Cao, Katarzyna J. Jerzak, Ipshita Prakash, Sandeep Sehdev, Christine Simmons, Nathaniel Bouganim, Muriel Brackstone, David W. Cescon, Stephen Chia, Ian S. Dayes, Scott Edwards, John Hilton, Anil Abraham Joy, Kara Laing, Marc Webster, Jan-Willem Henning

**Affiliations:** 1 Saskatchewan Cancer Agency, Regina, SK S4W 0G3, Canada; mita.manna@saskcancer.ca; 2 BC Cancer—Vancouver, Vancouver, BC V5Z 4E6, Canada; kgelmon@gmail.com (K.A.G.); christine.simmons@bccancer.bc.ca (C.S.); schia@bccancer.bc.ca (S.C.); 3 Jewish General Hospital, Montreal, QC H3T 1E2, Canada; boileaujf@gmail.com (J.-F.B.); ipshita.prakash@mcgill.ca (I.P.); 4 Mount Sinai Hospital, Toronto, ON M5G 1X5, Canada; christine.brezden@sinaihealth.ca; 5 Tom Baker Cancer Centre, Calgary, AB T2N 4N2, Canada; jeffrey.cao@albertahealthservices.ca (J.Q.C.); marc.webster@albertahealthservices.ca (M.W.); 6 Sunnybrook Health Sciences, Toronto, ON M4N 3M5, Canada; katarzyna.jerzak@sunnybrook.ca; 7 The Ottawa Hospital Cancer Centre, Ottawa, ON K1H 8L6, Canada; ssehdev@toh.ca (S.S.); jfhilton@toh.ca (J.H.); 8 McGill University Health Centre, Montréal, QC H4A 3J1, Canada; nbouganim@gmail.com; 9 St. Joseph’s Health Care London, London, ON M5G 1X5, Canada; muriel.brackstone@lhsc.on.ca; 10 Princess Margaret Cancer Centre, Toronto, ON M5G 2M9, Canada; dave.cescon@uhn.ca; 11 Juravinski Cancer Centre, Hamilton, ON L8V 5C2, Canada; dayes@hhsc.ca; 12 Dr. H. Bliss Murphy Cancer Center, St. John’s, NL A1B 3V6, Canada; scott.edwards@easternhealth.ca (S.E.); kara.laing@easternhealth.ca (K.L.); 13 Cross Cancer Institute, Edmonton, AB T6G 1Z2, Canada; anil.joy@albertahealthservices.ca

**Keywords:** HER2+, breast cancer, Canadian consensus recommendations, evidence-based, REAL Alliance

## Abstract

Human epidermal growth factor receptor 2-positive (HER2+) breast cancer is an aggressive subtype of breast cancer associated with a poor prognosis when sub-optimally treated. Recent advances include new and effective targeted therapies that have significantly improved outcomes for patients. Despite these advances, there are significant gaps across Canada, underscoring the need for evidence-based consensus guidance to inform treatment decisions. Addressing these gaps is crucial to ensuring that effective therapies are integrated into clinical practice, so as to improve the lives of patients affected by this aggressive form of breast cancer. The Research Excellence, Active Leadership (REAL) Canadian Breast Cancer Alliance is a standing nucleus committee of clinical-academic oncologists across Canada and Breast Cancer Canada, a patient organization. The mandate of this group is to provide evidence-based guidance on best practices in the management of patients with breast cancer. These consensus recommendations were developed using a modified Delphi process with up to three rounds of anonymous voting. Consensus was defined a priori as ≥75% of voters agreeing with the recommendation as written. There are 9 recommendations in the early setting; 7 recommendations in the metastatic setting; and 10 recommendations for patients with brain metastases.

## 1. Introduction

Breast cancer is the most prevalent cancer among women in Canada. It is estimated that more than 30,000 Canadian women will be diagnosed with breast cancer in 2024. It represents one-quarter of all new cancer cases and is the second leading cause of death from cancer in women [1].

Human epidermal growth factor receptor 2-positive (HER2+) breast cancer is characterized by amplification and/or overexpression of HER2 and is defined as HER2 immunohistochemistry (IHC) 3+ or IHC 2+/in situ hybridization (ISH)-amplified [2]. It is a particularly aggressive subtype that, without effective therapies, is associated with poor prognosis [3,4]. HER2+ breast cancer accounts for approximately 14% of breast cancer cases [4,5].

More than two decades of intensive clinical investigation have led to the development of multiple effective therapies that dramatically improve outcomes in HER2+ breast cancer. Despite these advancements in treatment, significant gaps remain in the current landscape across Canada. Evidence-based consensus guidance can help to inform treatment decisions and improve overall care delivery.

The Research Excellence, Active Leadership (REAL) Canadian Breast Cancer Alliance is a standing nucleus committee of clinical-academic oncologists across Canada and Breast Cancer Canada, a patient organization. The REAL Alliance was formed and launched in December 2023 due to the recognition that national collaboration is needed to address an unmet need: evidence-based guidance and recommendations, with timely updates, for use with public and government stakeholders for equitable and timely access to treatment and care for breast cancer patients. The aim of this paper—in line with the mission of the REAL Alliance—is to allow patients, advocates, health policy makers, and oncologists to work together to improve outcomes for patients with breast cancer across Canada.

## 2. Materials and Methods

### 2.1. Consensus Recommendation Process

A literature search was conducted to identify the evidence base relevant to the systemic treatment of HER2+ breast cancer, both in the early and metastatic setting (see Supplementary Materials). Three members of the REAL Alliance (M.M., K.A.G., J.W.H.) acted as the sub-committee and reviewed the studies to select the relevant clinical trials. The sub-committee also developed 19 preliminary recommendations related to systemic therapy for early and metastatic breast cancer, as well as treatment sequencing in patients with brain metastasis (BM). Relevant references were selected and linked to each recommendation.

These recommendations were then subjected to a modified Delphi process in which an initial round of anonymous voting was carried out by an expert panel using an electronic platform. This panel consisted of 14 medical oncologists, 3 surgical oncologists, 2 radiation oncologists, 1 oncology pharmacist, and 1 patient advocacy organization, all with specialized expertise and extensive experience in managing breast cancer. The participants represented several academic institutions and professional societies across Canada. During the voting process, panellists anonymously indicated their level of agreement with each statement, provided suggestions for revisions, and commented on specific references and background data. 

The statements were then revised accordingly and discussed further at a 2-day, in-person consensus meeting in Toronto, Ontario, Canada, in June 2024. During this meeting, the data were presented, and the wording of the recommendations was finalized. Following this meeting, a second round of anonymous electronic voting took place where participants evaluated their agreement with each statement after reviewing a summary of the discussion and the corresponding references. A third and final round of edits and anonymous voting was conducted. A recommendation was considered accepted if more than 75% of participants agreed with it after the predefined three rounds of voting. The strength of each recommendation was then determined by the consensus group according to the GRADE system for evidence quality assessment and strength-of-evidence recommendation [6]. A strong recommendation is based on evidence from a randomized, controlled trial in the intention-to-treat population and is considered standard of care. A strong consideration is based on a sub-population from a randomized controlled trial. A moderate recommendation is based on lower levels of evidence. Expert opinion is offered in situations where a clinical decision is required but there is limited evidence. As a final step, the sub-committee compared the current European Society of Medical Oncology (ESMO) and American Society of Medical Oncology (ASCO) guidelines to the REAL consensus recommendations.

### 2.2. Guiding Principles

At the outset of the development of these recommendations, the REAL Alliance agreed that due to the biology of breast cancer, it is essential to use the most efficacious therapies for both early and advanced disease. In the setting of early HER2+ breast cancer, the REAL Alliance recognized the shifting paradigm for optimal neoadjuvant treatment approaches, which may allow for escalating and/or de-escalating adjuvant therapies according to surgical outcomes.

Furthermore, even though these recommendations are arranged according to the line of therapy for advanced HER2+ breast cancer, clinical decision-making should be made according to the biology of the disease as well as patient factors (e.g., comorbidities, performance status, patient preferences and values) and should not be limited by strict adherence to predetermined sequencing restrictions. Including patient input with respect to preferences and values is an integral part of the decision-making process.

These consensus recommendations were developed based on the best available evidence, at the time of writing, without consideration of access or reimbursement constraints. The intention was that the application of these recommendations to Canadian practice will optimize outcomes for patients with HER2+ breast cancer.

Finally, it is noteworthy that enrollment in clinical trials is always encouraged for patients who meet the clinical trial eligibility criteria.

## 3. Systemic Therapy in HER2+ Breast Cancer

### 3.1. Early Breast Cancer

Early breast cancer is defined as stages I to III (see [7] for staging and the TNM definitions from the eighth edition of the American Joint Committee on Cancer [AJCC 8] staging manual), excluding those who present with stage IV or inoperable locally advanced breast cancer. This category includes people who have potentially curable breast cancer, albeit with different chances of cure. Those with operable locally advanced breast cancer (stage III cancers) have a much higher risk of relapse and ultimately death from breast cancer compared to those with lower-stage disease. Prior to 2005 and the commencement of use of trastuzumab, the prognosis of HER2+ early breast cancer was poor. Since then, with improved therapy for early-stage disease, prognosis has significantly improved. Data from the National Cancer Institute (NCI) suggest that more than 90% of individuals with early HER2+ breast cancer are alive at 5 years [8]. The improvement in survival is now leading to studies investigating de-escalation in early-stage HER2+ breast cancer, which is a clear indication of the efficacy of current treatments.

Although HER2 status is the focus of these recommendations, hormone receptor (HR) status (i.e., estrogen receptor [ER] and/or progesterone receptor [PR]) is also central to treatment decisions. For the purposes of these recommendations, if the patient is HR-positive (HR+), endocrine therapy (ET) should be started (along with ovarian suppression, if premenopausal) after chemotherapy and continued for at least 5 years or as per local protocols.

For those patients who receive neoadjuvant therapy, pathologic complete response (pCR)—the complete elimination of invasive disease in the breast and axillary lymph nodes—is an important intermediate outcome for early-stage HER2+ breast cancer. Experiencing pCR (or not) has strong implications for prognosis and subsequent adjuvant treatment. Patients who experience pCR have significantly improved long-term disease-free survival (DFS) compared to those harbouring residual disease, although the extent of residual disease also affects outcome. Indeed, residual invasive disease is the most significant adverse prognostic factor for DFS and overall survival (OS) in HER2+ breast cancer [9,10,11,12]; thus, it is important to identify these patients and escalate systemic therapy, as described in Recommendation 8. International efforts are being made to identify treatments that optimize pCR rates to improve the potential for long-term cure [11]. Notably, the importance of pCR and treatments that improve pCR rates has been incorporated into multiple European and American treatment and funding guidelines [11,13,14].

The REAL recommendations for early breast cancer are summarized in Table 1 and compared with ESMO and ASCO guidelines. Thereafter, the evidence supporting each recommendation is reviewed and summarized in Table 2.

**Recommendation** **1.** *For patients with HER2+ early breast cancer cT1a and b (i.e., ≤1 cm) without evidence of nodal disease (cN0), the standard of care is timely surgery followed by adjuvant treatment depending on the pathologic staging of disease (see Recommendations 3–5) (strong recommendation).*

The Adjuvant Paclitaxel and Trastuzumab (APT) trial was an open-label, single-arm, multicentre, phase 2 study that demonstrated the efficacy and safety of adjuvant paclitaxel and trastuzumab for node-negative, HER2+ breast cancer, with tumours ≤3 cm [15]. Among 406 patients, 49.5% had T1a or T1b tumours (≤1.0 cm), 41.6% had T1c disease (>1.0 to ≤2.0 cm), and 8.9% had T2 disease (>2.0 to ≤3.0 cm); the majority of tumours (67%) were hormone-receptor-positive (HR+) [15]. After a median follow-up of 10.8 years, 12 weeks of paclitaxel + trastuzumab followed by 9 months of trastuzumab monotherapy led to high rates of 10-year invasive disease-free survival (iDFS) (91.3%, 95% confidence interval [CI] 88.3–94.4), 10-year recurrence-free interval (RFI) (96.3%, 95% CI 94.3–98.3), and 10-year OS (94.3%, 95% CI 91.8–96.8) across the overall population [16]. Of note, many iDFS events and deaths were unrelated to the original breast cancer diagnosis [16]. The rate of serious toxicities was low [15].

The results of the APT trial established adjuvant paclitaxel and trastuzumab as the standard therapy in patients with small, node-negative HER2+ breast cancer.

This recommendation applies to node-negative disease, since the standard of care for node-positive disease—even in smaller tumours—is neoadjuvant therapy prior to surgery (discussed in subsequent sections) [13]. Furthermore, this recommendation is specifically for cT1a and b tumours (≤1 cm), because clinical node-negative disease may not equate to pathologic node negativity, particularly in larger tumours. A retrospective cohort study of 3333 women with stage I–II (cT1–T2) HER2+ breast cancer treated with upfront surgery without neoadjuvant therapy in 2018 demonstrated that tumour size was the strongest predictor of nodal positivity—both T1c and T2 disease were associated with increased nodal positivity with pathology [17]. With T1a and T1b disease (occurring in 30% of the cohort), 8.7% and 9.7%, respectively, were associated with nodal metastases; this increased to 22.7% with T1c and 36.9% with T2 tumours (occurring in 38% and 30% of the cohort, respectively) [17]. In single and multivariable analyses, T1a and b tumour sizes were not significantly associated with an increased risk of nodal metastases, while this was the case with T1c and T2 tumours. As such, cT1c tumours without evidence of nodal disease are considered in a separate recommendation (see Recommendation 2).

**Recommendation** **2.** *For patients with HER2+ early breast cancer cT1c (i.e., >1 to ≤2 cm) without evidence of nodal disease (cN0), the standard of care is surgery followed by adjuvant treatment (strong recommendation). However, due to current global practices, consideration can be given to neoadjuvant treatment followed by surgery and adjuvant treatment (strong consideration).*

As discussed above, patients with HER2+ T1N0 breast cancer have excellent outcomes with adjuvant therapy as per the APT regimen [16], but some patients with cT1 cN0 tumours may have pathologic node-positive disease [17]. In a multivariable analysis of a retrospective cohort study of 3333 women with stage I–II (cT1–T2) HER2+ breast cancer treated with upfront surgery without neoadjuvant therapy, T1c and T2 patients had a 3.4- and 6.3-fold increased incidence of node-positive disease relative to T1a patients (T1c odds ratio [OR]: 3.37, 95% CI 2.35–4.85; T2 OR: 6.34, 95% CI 4.41–9.10) [17]. Other predictors of nodal disease included younger age (<50 years) at diagnosis and estrogen receptor-positive (ER+) disease (triple-positive subtype in particular) [17].

Patients with T1c disease may benefit from neoadjuvant therapy, and subsequently should be assessed for evidence of residual disease after surgery. The KATHERINE trial—which enrolled patients with HER2+ early breast cancer with residual invasive disease at surgery after taxane-based neoadjuvant chemotherapy + trastuzumab-containing HER2 blockade—included 180 patients with cT1c tumours at diagnosis (>1 cm and ≤2 cm) [18]. Consistent with the overall trial outcomes, adjuvant trastuzumab emtansine (T-DM1) resulted in improved 3-year iDFS vs. adjuvant trastuzumab in the cT1c subpopulation (94.8% vs. 83.4%, hazard ratio [HR] 0.33, 95% CI 0.13–0.88) [18]. In the overall population at baseline, 77% had received a prior anthracycline treatment; as neoadjuvant HER2-targeted therapy, 80% had received trastuzumab alone, while 20% had received trastuzumab + pertuzumab or another HER2-targeted therapy [18]. In a subgroup analysis of 77 patients with cT1c cN0 tumours, 73% displayed prior anthracycline use, and 21% displayed prior pertuzumab use [19].

**Recommendation** **3.** *For patients with HER2+ early breast cancer with pT1 without evidence of nodal disease (pN0), the standard of care adjuvant systemic treatment is paclitaxel + trastuzumab for 12 weeks followed by trastuzumab monotherapy for 9 months (strong recommendation).*

The results of the APT trial established adjuvant paclitaxel and trastuzumab as the standard therapy in patients with small, node-negative HER2+ breast cancer, following surgery and locoregional radiotherapy, if indicated [15,16].

The weight of the evidence supports the APT regimen in these patients. The REAL Alliance recognizes other non-anthracycline regimes for this patient population, including docetaxel/carboplatin/trastuzumab (TCbH) [20] or docetaxel/cyclophosphamide/trastuzumab (DCH) [21].

**Recommendation** **4.** *Although neoadjuvant treatment is preferred, for those patients who are treated with upfront surgery and are then found to have ≥pT2 pN0 disease, the standard of care is adjuvant chemotherapy + trastuzumab (strong recommendation).*

Recognizing that neoadjuvant chemotherapy is preferred for patients with ≥T2 tumours, this recommendation refers to patients who, for whatever reason (e.g., patient preference, comorbid conditions, imaging/pathology discordance), had upfront surgery and were found to have ≥pT2 pN0 disease at surgery.

In line with Recommendation 3, the results of the APT trial established adjuvant paclitaxel and trastuzumab as the preferred standard therapy in patients with small (≤3 cm), node-negative HER2+ breast cancer following surgery and locoregional radiotherapy, if indicated. Additionally, the initial adjuvant studies included individuals with pT2N0 disease and demonstrated a benefit for adding trastuzumab to all the chemotherapy regimens studied, including anthracycline and non-anthracycline regimens, so these may be considered [20,22,23,24,25].

**Recommendation** **5.** *For patients with HER2+ early breast cancer with ≥cT2 or those with nodal disease (cN+), the standard of care is neoadjuvant therapy with trastuzumab + pertuzumab + chemotherapy (strong recommendation).*

This recommendation is a pivotal aspect of the early HER2+ Canadian consensus recommendations, as current practice often excludes pertuzumab due to a lack of public funding. Accepted practice for neoadjuvant therapy in early ≥cT2 or node-positive breast cancer is dual HER2 blockade with trastuzumab + pertuzumab (HP) + chemotherapy, with a consideration for using non-anthracycline-containing chemotherapy regimens for reduced toxicity, based on the safety and efficacy demonstrated across numerous phase 2 and 3 trials discussed below. Dual HER2 blockade with pertuzumab added to trastuzumab is complementary with respect to the mechanism of action [26,27]. Trastuzumab binds to the extracellular domain IV of the HER2 receptor, inhibiting HER2 dimerization, whereas pertuzumab binds to domain II inhibiting HER2 heterodimerization [26,27]. Neoadjuvant therapy with HP + chemotherapy improves rates of pCR and potentially avoids exposing patients to the morbidity associated with axillary lymph node dissection [28].

The NeoSphere study compared docetaxel + HP with docetaxel + trastuzumab (H) alone in operable (T2–3, N0–1, M0), locally advanced (T2–3, N2–N3, M0; T4a–c, any N, M0), or inflammatory (T4d, any N, M0) HER2+ breast cancer (N = 417). At baseline, 29% were node-negative, 45% had N1, 22% had N2, and 4% had N3 disease [29]. Docetaxel + HP led to significantly higher rates of pCR vs. docetaxel + H (45.8% vs. 29.0%, *p* = 0.0141) [29]. These results were consistent regardless of hormone receptor status, although pCR rates were lower overall in patients with HR+ disease [29]. Tolerability was similar across both arms, with few adverse events of grade 3 or higher [29]. After 5 years of follow-up, progression-free survival (PFS) was 86% with docetaxel + HP vs. 81% with docetaxel + H (HR 0.69, 95% CI 0.34–1.40, not powered for statistical comparison); there were no new or long-term safety concerns or differences in cardiotoxicity [30].

The phase 3 PEONY trial investigated the efficacy and safety of docetaxel + HP vs. docetaxel + H alone specifically in 329 Asian patients with HER+ early (T2–3, N0–1, M0) or locally advanced (T2–3, N2 or N3, M0; T4, any N, M0) breast cancer with a primary tumour size >2 cm [31]. Neoadjuvant docetaxel + HP resulted in significantly improved rates of pCR compared to docetaxel + H (39.3% vs. 21.8%, *p* = 0.001) [31]. The safety profile was similar between groups, with higher rates of diarrhea (mostly grade 1 and 2) and grade 3 neutropenia with dual HER2 blockade [31]. Following surgery, patients in the PEONY trial received adjuvant chemotherapy combined with either HP or H alone (consistent with the neoadjuvant regimen received). After 5 years of follow-up, neoadjuvant and adjuvant dual HER2 blockade, in addition to chemotherapy, led to increased rates of event-free survival (EFS, 84.8% vs. 73.7%; HR 0.53, 95% CI 0.32–0.89) and DFS (86.0% vs. 75.0%; HR 0.52, 95% CI 0.30–0.88) [32]. In an exploratory analysis, patients with pCR—regardless of the treatment arm—had increased 5-year DFS compared to patients without pCR (92.4% vs. 76.9%) [32].

BERENICE investigated the safety and efficacy of two neoadjuvant anthracycline-/taxane-containing regimens combined with dual HER2 blockade in cohorts of patients with early-stage, locally advanced, or inflammatory HER2+ breast cancer with normal cardiac function (n = 400). Cohort A received dose-dense doxorubicin plus cyclophosphamide (ddAC, four Q2W cycles) followed 2 weeks later by 12 QW paclitaxel (T) injections, while cohort B received 5-fluorouracil, epirubicin, and cyclophosphamide (FEC, four Q3W cycles), followed by docetaxel (D, four Q3W cycles). Both cohorts received neoadjuvant HP (four 3-week cycles) that continued for up to 1 year in the adjuvant setting. After a median follow-up of 14.5 months, pCR rates were 61.8% in cohort A and 60.7% in cohort B [33]. After a median follow-up of approximately 64 months in BERENICE, efficacy outcomes were similar in cohorts A and B (5-year EFS: 90.8% vs. 89.2%; 4-year iDFS: 92.6% vs. 91.1%; 5-year OS: 96.1% vs. 93.8%) [34]. Overall safety in BERENICE was consistent with anticipated profiles of the regimens; the most common serious AEs during neoadjuvant treatment were febrile neutropenia, neutropenia, and diarrhea [33].

In a retrospective analysis of 83 patients receiving neoadjuvant HER2 blockade between 2015 and 2021 at the Jewish General Hospital in Montreal, Quebec, dual HER2 blockade with HP (n = 39) was associated with more than double the pCR rate than with H alone (67% vs. 27%, *p* = 0.0016) [35]. These findings were consistent regardless of hormone receptor status. Given the reduction in the number of patients eligible for T-DM1 adjuvant therapy with improved pCR rates, the authors concluded that adding neoadjuvant P was cost-neutral [35].

Given the potential for cardiac dysfunction with trastuzumab—and, in particular, with anthracycline-containing chemotherapy—the TRYPHAENA study was designed to assess the safety and tolerability of neoadjuvant HP + anthracycline-containing chemotherapy vs. HP + anthracycline-free chemotherapy in 225 patients with HER2+ operable, locally advanced, or inflammatory breast cancer with primary tumours > 2 cm in diameter [36]. Patients received FEC (cycles 1–3) followed by docetaxel (cycles 4–6) with HP in all cycles; FEC (cycles 1–3) followed by docetaxel + HP (cycles 4–6); or docetaxel + carboplatin + HP for six cycles (i.e., anthracycline-free). After 5 years of follow-up, all regimens were generally well-tolerated, with low and similar rates of cardiac toxicity across arms [36]. During post-treatment follow-up, 9.2–13.3% of patients experienced adverse events, with only two serious adverse events (left ventricular systolic dysfunction and neutropenic infection, both in the FEC followed by docetaxel + HP arm) [36].

More recently, TRAIN-2 demonstrated no difference in rates of pCR (regardless of ER status) with neoadjuvant HP + non-anthracycline-containing chemotherapy vs. HP + anthracycline-containing chemotherapy in patients with stage II or III early breast cancer [37]. Importantly, the dual HER2 blockade + non-anthracycline-containing chemotherapy arm was associated with an improved safety profile, including significantly reduced rates of grade 4 neutropenia and grade 3 or 4 febrile neutropenia [37]. These results were consistent after 3 years of follow-up, with cardiotoxic effects and secondary malignant neoplasms also associated with anthracycline-based neoadjuvant chemotherapy [38]. Since the publication of the TRAIN-2 study, a non-anthracycline-containing chemotherapy regimen with dual HER2 blockade (i.e., TCbH+P) has become a preferred regimen in the neoadjuvant setting.

The consistent safety and tolerability findings across trials reinforce that the potential efficacy of adding neoadjuvant P to achieve dual HER2 blockade outweighs potential risks in appropriate HER2+ early breast cancer patients. While it is difficult to make recommendations about specific chemotherapy regimens since neoadjuvant regimens (and subsequent adjuvant treatment) differed across trials (see Table 2), the totality of the evidence supports the REAL recommendation that neoadjuvant chemotherapy + HP is the standard of care for patients with HER2+ early breast cancer with tumours ≥cT2 or those with node-positive disease (cN+).

**Table 2:** Neoadjuvant HER2+ early breast cancer trials: regimens and outcomes.

	Neoadjuvant Regimen *	Adjuvant Regimen	Primary Outcome	Secondary Outcomes
**NeoSphere [29,30]**	DHP (Q3W × 4) DH (Q3W × 4)	FEC (Q3W × 3)	pCR: 45.8% vs. 29.0%	5-year PFS: 86% vs. 81% (HR 0.69, 95% CI 0.34–1.40) 5-year DFS: 84% vs. 81% (HR 0.60, 95% CI 0.28–1.27)
**PEONY [31,32]**	DHP (Q3W × 4) DH (Q3W × 4)	FEC (Q3W × 3) followed by HP (13 cycles) FEC (Q3W × 3) followed by H (13 cycles)	pCR: 39.3% vs. 21.8% (Δ = 17.5%, 95% CI 6.9–28.0%, *p* = 0.001)	5-year EFS: 84.8% vs. 73.7% (HR 0.53, 95% CI 0.32–0.89) 5-year DFS: 86.0% vs. 75.0% (HR 0.52, 95% CI 0.30–0.88) 5-year OS: 93.9% vs. 90.0%(HR 0.53, 95% CI 0.23–1.19)
**BERENICE [33]**	ddAC (Q2W × 4) followed by T (QW × 12) + HP (Q3W × 4) FEC (Q3W × 4) followed by DHP (Q3W × 4)	HP (Q3W × 13)	NYHA class III/IV heart failure (patients): 3 (1.5%) vs. 0 Confirmed LVEF decline ^†^: 2 (1.0%) vs. 1 (0.5%)	5-year EFS: 90.8% vs. 89.2% 4-year iDFs: 92.6% vs. 91.1% 5-year OS: 96.1% vs. 93.8% pCR: 61.8% vs. 60.7%
**TRYPHAENA [36,39]**	FEC + HP (Q3W × 3) followed by DHP (Q3W × 3) FEC (Q3W × 3) followed by DHP (Q3W × 3) TCHP (Q3W x 6)	H (up to 1 year)	Symptomatic LVSD: 0 vs. 2 (2.7%) vs. 0 LVEF decline ^‡^: 4 (5.6%) vs. 4 (5.3%) vs. 3 (3.9%)	3-year DFS: 87% vs. 88% vs. 90% 3-year PFS: 89% vs. 89% vs. 87% 3-year OS: 94% vs. 94% vs. 93%
**TRAIN-2 [37,38]**	HP + FEC (Q3W × 3) followed by HP + TC (Q3W × 6) HP + TC (Q3W × 9)	N/A	pCR: 67% vs. 68% (Δ= −1.5%, 95% CI −11 to 8, *p* = 0.95)	3-year EFS: 92.7% vs. 93.6% (HR 0.90, 95% CI 0.50–1.63) 3-year OS: 97.7% vs. 98.2% (HR 0.91, 95% CI 0.35–2.36)

D, docetaxel; ddAC, dose-dense doxorubicin plus cyclophosphamide; FAC, 5-fluorouracil, doxorubicin, cyclophosphamide; T, paclitaxel; TC, paclitaxel + carboplatin; FEC, 5-fluorouracil, epirubicin, cyclophosphamide; TCHP, docetaxel, carboplatin, trastuzumab, pertuzumab; * NeoSphere had four treatment arms: group A (DHP); group B (DH); group C (PH without chemotherapy); and group D (PD). Only group A and B are of interest here. ^†^ At least 2 consecutive declines of ≥10% from baseline and to a value of <50% (symptomatic and asymptomatic) determined by means of echocardiography or multiple-gated acquisition scans. ^‡^ LVEF decline of ≥10% from baseline to <50%.

**Recommendation** **6.** *Although neoadjuvant treatment is preferred, for those patients who are treated with upfront surgery and are then found to have nodal disease in the pathological specimen (pN+), the standard of care is adjuvant chemotherapy + trastuzumab with consideration given to the addition of pertuzumab (strong consideration).*

This recommendation guides clinical decision-making in patients whose nodal disease is only recognized after surgery upon inspection of the pathological specimen. Dual HER2 blockade plus chemotherapy is the accepted standard of care for the neoadjuvant treatment of patients with HER2+ early breast cancer who have nodal disease, as discussed above (see Recommendation 5).

The APHINITY trial was conducted to explore the benefits of adding pertuzumab in the adjuvant setting. Patients in the APHINITY trial were enrolled after adequate excision of histologically confirmed HER2+ invasive breast cancer with either node-positive disease or high-risk, node-negative disease [40]. The primary end point of the trial was iDFS.

Across the 4805 patients assigned, 63% were node-positive and 64% had HR+ disease. With a 45-month median follow-up, invasive disease events were reported in 171 patients (7.1%) in the pertuzumab + trastuzumab + chemotherapy group and in 210 patients (8.7%) in the trastuzumab + chemotherapy group. The 3-year rate of iDFS in the intent-to-treat (ITT) population was 94.1% in the pertuzumab + trastuzumab + chemotherapy group and 93.2% in the trastuzumab + chemotherapy group HR of 0.81 (95% CI, 0.66 to 1.00; *p* = 0.045). Preplanned subgroup analysis revealed that there was no treatment effect in the patients with node-negative disease (HR, 1.13; 95% CI, 0.68–1.86; *p* = 0.64) and that patients with node-positive disease derived significant benefits (HR, 0.77; 95% CI, 0.62–0.96; *p* = 0.02) [40].

With a 74-month median follow-up, the node-positive cohort continued to demonstrate an iDFS benefit from pertuzumab + trastuzumab + chemotherapy compared to trastuzumab + chemotherapy with a 6-year iDFS of 88% and 83%, respectively (Δ = 4.5%; 95% CI, 1.9–7.1) [41]. As with the previous analysis, benefits were not seen in the node-negative cohort. The preplanned second interim OS did not reach statistical significance, requiring longer follow-up to fully assess the OS benefit.

Thus, the addition of pertuzumab to adjuvant trastuzumab in patients with node positive disease is a strong consideration.

**Recommendation** **7.** *For patients with HER2+ early breast cancer in whom a pathologic complete response is determined in the surgical specimen after completion of neoadjuvant trastuzumab and pertuzumab plus chemotherapy, the standard of care is trastuzumab for a total of 1 year (strong recommendation).*

Adjuvant trastuzumab alone is considered an appropriate therapy in the case of pCR following neoadjuvant chemotherapy + trastuzumab + pertuzumab. During the voting process, some members of the REAL Alliance stated that they sometimes consider adding pertuzumab to trastuzumab in the adjuvant setting.

As stated in Recommendation 5, the standard of care in Canada for patients with ≥cT2 disease or those with node-positive disease is neoadjuvant trastuzumab + pertuzumab + chemotherapy.

APHINITY investigated the role of dual blockade in the adjuvant setting in HER2+ early breast cancer with node-positive disease or node-negative disease with tumours > 1.0 cm in patients who were chemotherapy- and anti-HER2-antibody-naïve (i.e., no neoadjuvant therapy) [40]. Adding pertuzumab to trastuzumab + chemotherapy increased 3-year iDFS vs. chemotherapy + trastuzumab alone (94.1% vs. 93.2%; HR 0.81, 95% CI 0.66–1.00, *p* = 0.045); however, no treatment effect was observed in the prespecified subgroup of patients with node-negative disease (HR 1.13, 95% CI 0.68–1.86, *p* = 0.64) [40]. The absence of a treatment effect of dual HER2 blockade in the node-negative (tumours > 1.0 cm) subgroup of the APHINITY suggests that trastuzumab alone is sufficient in the adjuvant setting [40].

The recommendation for the 1-year duration is based on findings from the HERA trial [24]. In HERA, patients with HER2+ early invasive breast cancer who underwent surgery (with or without radiotherapy) plus adjuvant and/or neoadjuvant chemotherapy and had node-positive disease or node-negative disease with tumours > 1 cm were randomized to adjuvant trastuzumab for 1 year, 2 years, or observation [24]. After 11 years of follow-up, disease-free survival was similar for 1- and 2-year adjuvant treatment arms, and was superior to observation (HR vs. observation, 1-year: 0.76, 95% CI 0.68–0.86; 2-year: 0.77, 95% CI 0.69–0.87; ITT analysis) [25]. Overall survival after 12 years was also similar in both trastuzumab groups.

Taken together, after neoadjuvant chemotherapy + trastuzumab + pertuzumab and confirmed pCR, adjuvant trastuzumab for a total of 1 year is the standard of care.

**Recommendation** **8.** *For patients with HER2+ early breast cancer in whom residual invasive disease is detected pathologically in the surgical specimen of the breast or axillary lymph nodes after completion of neoadjuvant trastuzumab + pertuzumab + chemotherapy, the standard of care is to treat with trastuzumab emtansine (T-DM1) for 14 cycles in the adjuvant setting (strong recommendation).*

In patients who have residual disease post-surgery, the risk of disease recurrence or death is higher than in those with pCR [10,30]. The KATHERINE trial investigated the efficacy and safety of adjuvant T-DM1 vs. trastuzumab in these higher-risk patients, enrolling 1486 patients with HER2+ early breast cancer with residual invasive disease at surgery after neoadjuvant chemotherapy + trastuzumab (additional HER2-targeted agents were permitted) [18]. At a 3-year follow-up, iDFS was significantly higher with adjuvant T-DM1 vs. trastuzumab (88.3% vs. 77.0%; HR 0.50, 95% CI 0.39–0.64; *p* < 0.001) [18].

Risk of distant metastases was lower with T-DM1 and consistent across subgroups, including hormone receptor status and neoadjuvant HER2-targeted treatment [18].

Adjuvant T-DM1 toxicity included higher rates of grade ≥3 adverse events (AEs) and serious AE (12.7% T-DM1, 8.1% trastuzumab) [18]. There was a higher discontinuation rate in the T-DM1 arm (18% vs. 2.1%), with the most common AE leading to discontinuation consisting of laboratory abnormalities (thrombocytopenia, transaminitis/hyperbilirubinemia), peripheral sensory neuropathy, and decreased ejection fraction.

Of 133 patients who discontinued T-DM1, 53.4% switched to trastuzumab and the majority completed 14 cycles of adjuvant HER2-directed therapy [18].

Adjuvant radiotherapy should be given according to standard guidelines and can be administered concurrently with adjuvant HER2-targeted therapy. In the KATHERINE study, patients presenting with cT3 node-positive disease or T4 disease and/or with cN2 or N3 disease received regional node radiotherapy with whole-breast or chest irradiation; this approach was also recommended for patients with T3N0 with residual nodal disease [18]. Otherwise, radiotherapy was administered as per institutional standards. Furthermore, in KATHERINE, ET was administered concurrently with HER2-directed therapy in patients with HR+ disease.

**Recommendation** **9.** *Although there is not a survival benefit, for patients with HER2+ HR+ and N+ disease who have completed (neo)adjuvant chemotherapy + trastuzumab, extended adjuvant treatment with neratinib for 1 year after completion of trastuzumab-based adjuvant therapy can be considered to decrease recurrence (moderate recommendation).*

ExteNET enrolled patients with HER2-positive, histologically confirmed stage II-IIIc operable breast cancer. After completing up to 1 year of neoadjuvant and adjuvant trastuzumab, 2840 patients were randomized to receive neratinib or a placebo for up to 1 year. After a median follow-up of 5.2 years, neratinib significantly extended iDFS vs. the placebo, including in patients with high-risk node-positive disease (HR 0.70, 95% CI 0.54–0.92, *p* = 0.010). The hypothesis-generating subgroup analysis favoured patients with HR+ disease (HR 0.60, 95% CI 0.43–0.83), but not HR– disease (HR 0.95, 95% CI 0.66–1.35) [42].

The treatment effect was present in patients with node-positive disease, who made up the majority of the study population; node-positive, HR+ patients who had received neratinib treatment within 1 year of completing adjuvant trastuzumab had extended central nervous system (CNS) DFS vs. placebo (98.2% vs. 95.4%, HR 0.41, 95% CI 0.17–0.89) [43]. Serious AE of diarrhea, nausea, and dehydration were more common with neratinib during treatment, but neratinib was not associated with long-term or late-occurring toxicities [42,43].

Neratinib can be given to reduce recurrence risk in high-risk patients, but no OS benefit was shown in ExteNET. Estimated 8-year OS rates were 91.5% with neratinib vs. 89.4% with placebo; OS was not significantly extended (HR 0.79, 95% CI 0.55–1.13) [43]. An important consideration is that the ExteNET trial predated our contemporary neoadjuvant and adjuvant therapies used in HER2-positive early breast cancer.

**Figure 1:**
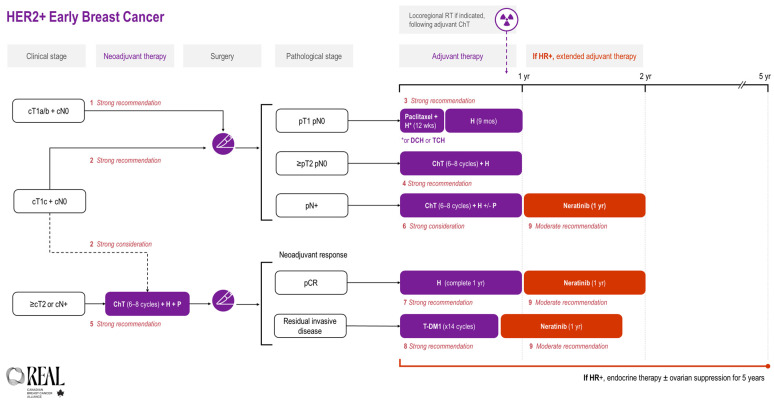
REAL Alliance recommendations for the treatment of HER2+ early breast cancer. ChT, chemotherapy; cN0, no nodal disease based on clinical assessment; CR, complete response; cT1a/b, tumour ≤ 1 cm on clinical assessment; cT1c, tumours > 1 cm but ≤2 cm on clinical assessment; cT2, tumours > 2 cm but ≤5 cm on clinical assessment; DCH, docetaxel + cyclophosphamide + trastuzumab; H, trastuzumab; HR, hormone receptor; p, based on pathologic assessment; P, pertuzumab; RT, radiotherapy; TCH, docetaxel + carboplatin + trastuzumab.

All REAL Alliance recommendations for early breast cancer are outlined in an algorithm (see Figure 1).

### 3.2. Metastatic Breast Cancer

The REAL recommendations for metastatic breast cancer are summarized in Table 3 and compared with ESMO and ASCO guidelines. Thereafter, the evidence supporting each recommendation is reviewed.

**Recommendation** **10.** *When safe and feasible, repeat biopsy should be performed in all patients whose disease relapses on or after adjuvant treatment (strong recommendation).*

Breast cancer is a heterogeneous disease, even within the various subtypes. Obtaining a biopsy at the time of disease relapse allows for re-confirmation of ER, PR, and HER2 status. Tissue biopsy is the current practice, though liquid biopsy may soon be an additional option and may also be used to direct therapy for precision oncology.

**Recommendation** **11.** *For patients with de novo HER2+ (HR±) metastatic breast cancer who have not received prior HER2-directed therapy or chemotherapy for metastatic disease or with disease that relapses >6 months after completion of (neo)adjuvant chemotherapy + HER2-directed therapy, the standard of care is trastuzumab + pertuzumab + taxane chemotherapy followed by trastuzumab + pertuzumab +/− ET maintenance therapy (strong recommendation).*

The phase 3 CLEOPATRA trial established dual HER2-targeted therapy (trastuzumab + pertuzumab) with docetaxel as the standard first-line treatment for HER2+ metastatic disease [44]. In the final analysis, this three-drug combination improved median OS by more than 16 months compared to trastuzumab + docetaxel alone (57.1 months vs. 40.8 months; HR 0.69, 95% CI 0.58–0.82). After 8 years of follow-up, the pertuzumab group had an OS rate of 37% (versus 23% in the placebo group), with 16% remaining progression free. Long-term safety was maintained [44].

Docetaxel was administered for 6 cycles or longer at the investigator’s discretion. Following this protocol, in the absence of disease progression, chemotherapy could be omitted after best tumour response was achieved (approximately six to eight cycles of chemotherapy), with trastuzumab + pertuzumab continued as maintenance therapy. “Best response” was defined as a complete response (CR), partial response (PR), or stable disease based on imaging or physical exams (clinical or radiographic). Additional patient factors to consider when deciding when to switch to maintenance therapy are the symptom burden, toxicities, quality of life, and preference. Consistent benefit was seen across subgroups, including those who received prior (neo)adjuvant systemic treatment including trastuzumab [45].

At the 8-year landmark evaluation, the OS benefit of pertuzumab in the prior trastuzumab subgroup was consistent with that seen in the overall population (median OS, 53.8 months vs. 46.6 months; HR 0.86, 95% CI 0.51–1.43 in a post hoc analysis). Patients who had received any previous (neo)adjuvant systemic therapy also saw consistent OS benefit with the pertuzumab regimen (HR, 0.74, 95% CI 0.57–0.95 vs. HR 0.65, 95% CI 0.51–0.82 in those with no prior (neo)adjuvant systemic therapy) [44].

Approximately half of the 808 patients enrolled in CLEOPATRA were HR+. The recommended treatment showed similar PFS and OS benefits, regardless of HR status. Of note, the trial did not allow for concurrent maintenance ET; however, maintenance ET has been widely adopted as common practice for HR+ disease [44,45].

The single-arm, multicentre PERUSE study confirmed and extended the CLEOPATRA findings, showing that paclitaxel is a suitable alternative to docetaxel in a similar first-line metastatic setting [46].

The phase 2 PERTAIN trial (258 patients) showed that for patients with HER2+, HR+ metastatic or locally advanced breast cancer, dual HER2-targeted therapy offers a significant PFS benefit compared to trastuzumab alone when combined with standard endocrine therapy (an aromatase inhibitor) (18.9 months vs. 15.8 months; HR 0.65, 95% CI, 0.48–0.89, *p* = 0.007) [47]. Patients were allowed up to 24 weeks of induction taxane chemotherapy prior to ET at the investigator’s discretion. PERTAIN confirmed that the combination of pertuzumab + trastuzumab + ET has an acceptable tolerability profile, consistent with previous trials. No new safety signals were reported with the addition of an aromatase inhibitor [47].

**Recommendation** **12.** *For patients with HER2-positive (HR±) metastatic breast cancer whose disease relapses ≤6 months after completion of (neo)adjuvant chemotherapy + HER2-directed therapy, the recommended treatment is as per the second-line recommendation (see Recommendation 13) (strong recommendation).*

**Recommendation** **13.** *For patients with HER2-positive metastatic breast cancer whose disease has progressed on first-line HER2-directed therapy, the standard of care is trastuzumab deruxtecan (T-DXd) in the absence of contraindications (strong recommendation).*

The antibody–drug conjugates (ADCs) trastuzumab deruxtecan (T-DXd) and T-DM1 each consist of an anti-HER2 monoclonal antibody linked to a cytotoxic payload [48]. DESTINY-Breast03 established T-DXd as the preferred second-line treatment for HER2-positive metastatic breast cancer, replacing T-DM1 as the standard of care in eligible patients.

The open-label, phase 3 DESTINY-Breast03 trial enrolled patients with unresectable or metastatic HER2-positive breast cancer who had received prior anti-HER2 treatment in the metastatic setting, as well as individuals who had progressed during or within 6 months of trastuzumab + taxane therapy in the (neo)adjuvant setting. Patients were randomized 1:1 to receive either T-DXd or T-DM1 [49].

The DESTINY-Breast03 trial provided direct evidence that T-DXd had superior efficacy to T-DM1 in the second-line metastatic setting. Enrolled patients had previously received trastuzumab + taxane, and approximately 60% had also received pertuzumab. T-DXd significantly extended median PFS (28.8 months vs. 6.8 months; HR 0.33, 95% CI 0.26–0.43; *p* < 0.0001) and improved the 24-month OS rate (77.4% vs. 69.9%). These benefits were consistent across subgroups stratified by hormone receptor status, previous treatment with pertuzumab, and history of visceral disease [49]. In a long-term survival analysis (median follow-up, 41 months), T-DXd maintained a manageable safety profile and superior efficacy over T-DM1, providing an OS benefit of almost 10 months (median OS, 52.6 vs. 42.7 months; HR 0.73, 95%CI 0.65–9.94) [50]. Endocrine therapy is not continued for HR+ in the second-line metastatic setting.

Earlier studies established T-DM1 as an efficacious and tolerable treatment for HER2-positive metastatic breast cancer refractory to prior HER2-targeted treatment. When T-DXd is contraindicated, T-DM1 remains a preferred option provided that it is not also contraindicated.

A notable AE with T-DXd is the risk of interstitial lung disease (ILD)/pneumonitis, which can be fatal if untreated. In DESTINY-Breast03, adjudicated drug-related ILD/pneumonitis occurred in 15% of patients treated with T-DXd compared to 3% of patients treated with T-DM1 [49].

Asymptomatic (grade 1) ILD can be monitored and treated effectively, allowing T-DXd treatment continuation. However, T-DXd must be immediately and permanently discontinued for ILD of grade 2 or higher. A pooled analysis of nine T-DXd monotherapy trials found that baseline or prior lung comorbidities (asthma, chronic obstructive pulmonary disease, prior ILD/pulmonary fibrosis, and radiation pneumonitis), renal dysfunction, age < 65 years, T-DXd dose > 6.4 mg/kg (which is above the usual breast cancer dose), and a drop in blood oxygen levels to <95% during therapy were associated with an increased risk of T-DXd-related ILD [51]. However, since many ILD cases are not associated with identifiable pre-existing risk factors, vigilant monitoring is required for all patients receiving T-DXd. Canadian practice guidelines from Henning et al. recommend a multidisciplinary approach to managing T-DXd-related ILD, involving medical oncologists, radiologists, respirologists, and allied health care professionals (https://pubmed.ncbi.nlm.nih.gov/37754497/, accessed on 20 September 2024) [52].

**Recommendation** **14.** *For patients with HER2+ metastatic breast cancer whose disease has progressed after at least 2 HER2-directed therapies the recommendation for treatment is tucatinib + capecitabine + trastuzumab (can be considered earlier if brain metastasis is present) (moderate recommendation).*

Tucatinib is an oral tyrosine kinase inhibitor with high selectivity for HER2. HER2CLIMB included 612 adults with HER2+ advanced breast cancer who had previously been treated with trastuzumab, pertuzumab, and T-DM1. Patients were randomized to receive either tucatinib or a placebo, in combination with trastuzumab and capecitabine. Estimated PFS at 1 year was 33.1% with the tucatinib combination vs. 12.3% with the placebo combination, with risk of progression or death reduced by 46% (HR 0.54, 95% CI 0.42–0.71, *p* < 0.001) [53]. Overall survival at 2 years was extended with the tucatinib combination (44.9% vs. 26.6% with placebo combination; HR 0.66, 95% CI 0.50–0.88, *p* = 0.005) [53]. These findings were consistent across subgroups.

If BM is present, tucatinib + capecitabine + trastuzumab can be considered earlier. Patients with BM were included in HER2CLIMB and comprised just under 50% of the study population. The results in patients with BM were consistent with the overall study population. Estimated 1-year PFS was 24.9% with the tucatinib combination vs. 0% in the placebo combination group (HR 0.48, 95% CI 0.34–0.69, *p* < 0.001) [53]. Brain metastases are discussed in detail in Section 3.3.

Diarrhea was the most common AE in both groups and was mostly grade 1 or 2, transient, and reversible. The most common AEs of grade ≥3 with the tucatinib combination were palmar–plantar erythrodysesthesia syndrome, diarrhea, ALT and AST elevations, and fatigue. AEs led to discontinuation of tucatinib in 5.7% of patients vs. 3.0% in the placebo group [53].

Outcomes in patients who have received at least two HER2-directed therapies are generally poor and clinical trials should be considered. There are many ongoing studies with newer-generation agents offering HER2 blockade or combinations of ADCs, which may be options and may provide access to new therapies.

**Recommendation** **15.** *For patients with HER2+ metastatic breast cancer whose disease has progressed after at least three HER2-directed therapies, the recommendation based on evidence is to continue HER2-directed therapy. Options include T-DXd (preferred option, if not previously used); chemotherapy + trastuzumab or another monoclonal antibody; T-DM1; neratinib +/− capecitabine; and lapatinib + capecitabine (moderate recommendation).*

Treatment for patients with HER2+ metastatic breast cancer that has progressed after at least three HER2-directed therapies should continue HER2-directed therapy, but the regimen should be selected based on patient and physician preference, such as the toxicity profile or previous exposure. Options included in this recommendation are listed in no order. Patients should receive all available evidence-based HER2-directed therapeutic classes throughout the disease trajectory. Furthermore, sequencing of therapies should be prioritized based on evidence and patient characteristics.

The exception to this is if a patient has not had previous T-DXd exposure, in which case T-DXd is preferred [49,54,55]. Given the remarkable activity of T-DXd in DESTINY-Breast02 and DESTINY-Breast03, patients who have not received T-DXd in an earlier line of treatment and who have no contraindications should receive it. T-DXd can be considered following tucatinib + capecitabine + trastuzumab if it has not already been used.

DESTINY-Breast02 was an open-label, multicentre, phase 3 trial [55]. It demonstrated that T-DXd was superior to conventional chemotherapy-based combinations with HER2-targeting agents—either capecitabine + trastuzumab or capecitabine + lapatinib—in HER2-positive metastatic breast cancer previously treated with T-DM1. Among 608 patients, T-DXd significantly improved both median PFS (17.8 months vs. 6.9 months; HR 0.36, 95% CI 0.28–0.45; *p* < 0.0001) and median OS (39.2 months vs. 26.5 months; HR 0.66, 95% CI 0.50–0.86; *p* = 0.0021). This trial was the first to show in a randomized setting that one ADC could overcome resistance to another. In DESTINY-Breast02, 47% of patients had three or more lines of prior therapy. Outcomes were consistent in the subgroup of patients who had received at least three lines of prior therapy, with a median PFS of 18.2 months with T-DXd vs. 6.9 months with treatment of physician’s choice (HR 0.41, 95% CI 0.29–0.57) [55].

In DESTINY-Breast03 (also reviewed in Recommendation 13), ~35% of patients had three or more lines of therapy. Subgroup analyses in patients who had at least three lines of previous therapy demonstrated a similar benefit to the overall trial (median OS not estimable for T-DXd vs. 22.8 months for T-DM1; HR 0.55, 95% CI 0.34–0.89) [49]. In DESTINY-Breast01, the median number of previous lines of therapy was 6 (range of 2–27). Among the 184 patients who received 5.4 mg/kg T-DXd, median PFS was 19.4 months (95% CI, 14.1–25.0; median follow-up 26.5 months) [56].

Evidence supporting the use of T-DM1 in later lines of therapy is based on findings from TH3RESA and EMILIA, which also included subgroups of patients who received at least three prior lines of systemic therapy for metastatic disease [57,58]. The phase 3 TH3RESA trial compared T-DM1 to the physician’s choice of treatment in a heavily pretreated population and demonstrated a consistent OS effect among the 65% of patients who had received more than three advanced breast cancer regimens, with a median OS of 21.6 months with T-DM1 vs. 15.5 months with the physician’s choice (HR 0.65, 95% CI 0.50–0.86) [57]. Subgroup analyses of EMILIA demonstrated similar benefits in those with two or more prior treatments compared to the overall population (HR for progression or death 0.69, 95% CI 0.55–0.86) [58].

Other chemotherapy + HER2-targeted therapy combinations may be used, including chemotherapy + trastuzumab (or another monoclonal antibody, e.g., margetuximab), neratinib +/− capecitabine, or lapatinib + capecitabine. Key trials supporting these options are summarized in Table 4.

Finally, investigators of the monarcHER clinical trial demonstrated a DFS benefit using a chemotherapy-free regimen, abemaciclib + HER2-directed therapy, compared to chemotherapy + HER2-directed therapy [59]. This may be an option for patients who are ineligible for chemotherapy-based anti-HER2 and ADC regimens in the fourth line or beyond.

**Table 4:** Summary of trials of other regimens for third+ regimen in metastatic breast cancer.

	Study Population	Key Outcomes (Intervention vs. Comparator Arm), HR (95% CI)	Key Safety Observations
**DESTINY-Breast01 [54]** Phase 2 T-DXd	HER2+ unresectable or mBC with prior T-DM1 (N = 184)	Median PFS: 19.4 months (14.1–25.0) Median OS: 29.1 months (24.6–36.1)	Grade ≥3 TEAE occurred in 53.8% of patients Most common TEAEs GI or hematologic Drug-related ILD/pneumonitis in 15.8%
**NALA [60]** Phase 3 Neratinib + capecitabine (+loperamide prophylaxis) vs. lapatinib + capecitabine	HER2+ mBC and ≥ 2 previous HER2-directed mBC therapies (N = 621)	PFS: 0.76 (0.63–0.93) OS: 0.88 (0.72–1.07)	Diarrhea, nausea, PPES, vomiting most common AEs of any grade overall More grade 3 diarrhea with neratinib No new cardiac safety concerns
	Subgroup with ≥3 previous HER2-targeted regimens: 30%	PFS: 0.71 (0.50–1.00) OS: 0.71 (0.50–1.02)	
**NCT00078572 [61]** Phase 3 Lapatinib + capecitabine vs. capecitabine alone	HER2+ locally advanced or mBC and progression after anthracycline, taxane, and H (N = 324) Prior H for mBC: 91%	PFS: 0.49 (0.34–0.71) OS: 0.92 (0.58–1.46)	Diarrhea, dyspepsia, rash, asymptomatic cardia events more common with lapatinib
**SOPHIA [62]** Phase 3 Margetuximab vs. H, each in combination with chemotherapy (1 of capecitabine, eribulin, gemcitabine, or vinorelbine)	HER2+ mBC, ≥2 previous HER2-directed therapies, and 1–3 nonhormonal mBC therapy (N = 536)	PFS: 0.76 (0.59–0.98) OS: 0.89 (0.69–1.13)	Grade ≥3 AEs (≥5%): neutropenia, anemia (both groups); fatigue (margetuximab); febrile neutropenia (H) ↑IRRs with margetuximab; no increase in cardiac toxicity
	Subgroup with ≥3 previous mBC regimens: 34%	PFS: 0.72 (0.48–1.08) OS: 0.70 (0.47–1.05)	

AE, adverse event; CI, confidence interval; GI, gastrointestinal; H, trastuzumab; HR, hazard ratio; IRR, infusion-related reaction; mBC, metastatic breast cancer; OS, overall survival; PFS, progression-free survival; PPES, palmar-plantar erythrodysesthesia syndrome.

**Figure 2:**
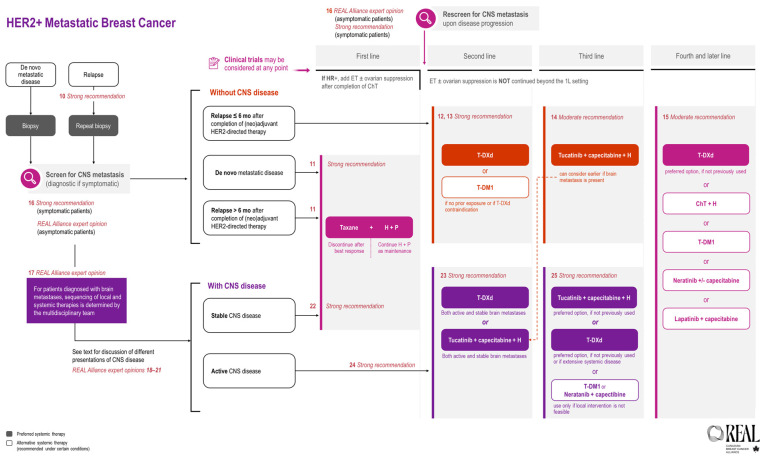
REAL Alliance recommendations for the treatment of metastatic breast cancer and BM.

All REAL Alliance recommendations for metastatic breast cancer are outlined in an algorithm (see Figure 2).

### 3.3. Brain Metastases

Patients with HER2+ metastatic breast cancer are two to four times more likely to develop CNS metastases than patients with HER2– metastatic disease [63]. In a retrospective French ESME database study (N = 16,703 patients treated first-line for metastatic breast cancer between January 2008 and December 2014), cumulative incidence rates of CNS metastases 24 months after metastatic breast cancer diagnosis were 29.2% and 49.0% in patients with HER2+/HR+ and HER2+/HR− disease, respectively [64]. In a systematic review and meta-analysis, the cumulative incidence of BM was 31% after a median follow-up of 30.7 months in patients with HER2+ breast cancer [65]. In an Ontario population-based cohort study, among 3916 patients with metastatic breast cancer, cumulative incidence of BM receiving radiation therapy after 3 years was 17.7% for patients with HER2+/HR+ disease and 25.3% in HER2+/HR− disease [66].

Among the patients in the KATHERINE trial who experienced distant recurrence, 36% of recurrences were in the brain [67]. Patients with metastatic breast cancer who develop BM have a poor prognosis. The registHER study documented that the median survival in women with HER2+ metastatic breast cancer who developed CNS metastases was 26.3 months compared to 44.6 months among those who did not develop BM [68]. After a median follow-up of 42.8 in the French ESME database, patients with BM had a median OS of 7.9 months and a neurological PFS of 5.5 months; HER2+ HR− tumours were associated with a worse PFS [69]. Furthermore, BM are increasingly seen as the first site of relapse [44].

Recommendations may refer to stable or active BM. Based on regulatory guidance for cancer clinical trial eligibility criteria, stable is defined as treated with local modalities and non-progressive; active is defined as either untreated asymptomatic or treated with local modalities but progressive [70].

Leptomeningeal metastasis is a subtype of CNS metastases with a very poor prognosis and limited evidence [71]. A phase 2 single-arm study recently demonstrated an OS benefit with trastuzumab, tucatinib, and capecitabine [72]. Of note, there is increasing interest in craniospinal irradiation with advances in radiation treatment delivery [73]. In general, the REAL recommendations apply to parenchymal disease. While data are limited, systemic therapy recommendations should follow those for other CNS disease scenarios.

The REAL recommendations for brain metastases are summarized in Table 5 and compared with ESMO and ASCO guidelines. Thereafter, the evidence supporting each recommendation is reviewed.

**Recommendation** **16.** *For patients with HER2+ metastatic breast cancer who have symptoms suggestive of CNS metastases, appropriate diagnostic investigations for CNS metastases are essential (strong recommendation). For patients with HER2+ metastatic breast cancer, screening for CNS metastases should be considered in asymptomatic patients at baseline in the metastatic setting and at disease progression (REAL Alliance Expert opinion).*

For patients with symptoms suggestive of CNS disease, such as new-onset headaches, unexplained nausea or vomiting, or changes in motor or sensory function, ASCO, ESMO, and NCCN guidelines all recommend performing diagnostic brain magnetic resonance imaging (MRI) testing [14,74,75]. The REAL Alliance endorses these recommendations.

In asymptomatic patients, the current ASCO Guidelines state that “there are insufficient data to recommend for or against performing routine surveillance” [74]. Notably, the ASCO Expert Panel changed this recommendation from recommending against routine screening to stating that there is insufficient evidence considering the emerging evidence in this area. The current ESMO Guidelines acknowledge the higher rates of brain metastases at initial diagnosis of metastatic disease in asymptomatic patients with HER2+ breast cancer or TNBC; thus, the guideline states that subtype-oriented brain imaging may be warranted in asymptomatic patients with metastatic disease “if presence of CNS metastases will alter the choice of systemic therapy” [75].

Kim et al. prospectively enrolled patients newly diagnosed with metastatic HER2+ breast cancer (and TNBC) between 2018 and 2023 [76]. Screening brain MRI was performed at diagnosis and after the failure of first- and second-line systemic therapy for all patients. Any patient who developed symptoms of BM and who was then diagnosed with BM was dropped from the study and followed for outcomes as the symptom-detected group. In the HER2+ surveillance-detected group, BM was present in 8.9% of patients at diagnoses and in 26.7% throughout treatment. These are patients who had no symptoms; thus, the BM would not have been detected without surveillance. The median PFS in the surveillance-detected group was 20.3 months compared to 7.6 months in the symptomatic-detected group [76].

As an expert group, the REAL Alliance recommends screening for CNS disease in asymptomatic patients at the time of diagnosis of metastatic HER2+ disease and/or at the time of disease progression as the presence of CNS metastases affects morbidity and mortality and may change the systemic treatment plan and allow for stereotactic radiosurgery (SRS) before the development of life-altering CNS symptoms. The REAL Alliance fully supports multidisciplinary tumour discussions for all patients with CNS disease, as noted in Recommendation 17, to maximize the benefits to patients of preventing or delaying CNS symptoms.

**Recommendation** **17.** *For patients with a history of HER2+ metastatic breast cancer who are diagnosed with brain metastases, multidisciplinary care with representation from radiology, radiation oncology, neurosurgery, medical oncology, and supportive care is the standard of care, with the multidisciplinary team (MDT) providing recommendations on sequencing of local and systemic therapies (REAL Alliance Expert opinion).*

Patients with HER2+ metastatic breast cancer who are diagnosed with BM have shorter survival and reduced quality of life compared to patients without BM [68,71] The primary treatment for such patients includes surgical excision and/or radiation therapy [77]. Thus, a team approach is required to determine the best way to sequence and prioritize therapies [78].

**Recommendation** **18.** For patients with a history of HER2+ metastatic breast cancer but without evidence of other extracranial systemic disease who present with oligometastatic brain disease amenable to local therapy, there is insufficient evidence to make a recommendation for a change in systemic therapy. Multidisciplinary care* is the standard of care, and the MDT is to make recommendations on sequencing of local and systemic therapies in such patients (REAL Alliance Expert opinion). *Includes radiologist (neuroradiologist, if available), radiation oncologist, neurosurgeon, medical oncologist, and supportive care.

The European Society of Radiotherapy and Oncology (ESTRO) and the American Society of Radiation Oncology (ASTRO) define oligometastatic disease as one to five metastatic lesions where all metastatic sites are “safely treatable” with or without disease control of the primary tumour [79]. This imaging-based definition does not take into account any patient characteristics, such as the disease-free interval (DFI) or HR status [80]. With the rapidly changing evidence landscape and many ongoing clinical trials, the decision of how best to sequence treatments and when to prioritize local treatments over systemic treatments must be made on a case-by-case basis. Thus, the REAL Alliance recommends multidisciplinary care in patients with oligometastases to decide on the appropriate sequence of treatments.

**Recommendation** **19.** *For patients with a history of HER2+ metastatic disease who present with asymptomatic, low-volume, newly diagnosed brain metastases, treatment should be discussed by the MDT incorporating patient values with treatment options including initial HER2-directed systemic therapy versus upfront local therapy (REAL Alliance Expert Opinion).*

Local therapy, either with surgery or radiotherapy, has been the mainstay of management for patients with BM. Recently, a deferral of local therapy in favour of systemic treatment has been gaining momentum. As noted in the most recent ASCO update, the patients best suited to such an approach are those without symptomatic mass effects, with a small single lesion (i.e., <2 cm) or, if multiple lesions, then a low bulk of disease (i.e., <2 cm) [74].

The REAL Alliance recommends discussing these patients at MDT rounds. The selection of HER2-directed systemic therapy is guided by HER2CLIMB or DESTINY-Breast03. These trials are discussed in Recommendations 22 and 23.

TUXEDO-1, a single-arm phase II trial, also evaluated T-DXd in 15 patients with HER2+ metastatic breast cancer and active BM. At a 26.5-month median follow-up, the trial reported the final results of a median PFS of 21 months and a median OS not reached, indicating a high intracranial response rate [81].

**Recommendation** **20.** *For patients with a history of HER2+ metastatic disease who present with symptomatic, newly diagnosed BM, upfront stereotactic radiosurgery (SRS) is a reasonable approach when technically feasible (and often preferred over whole brain radiotherapy [WBRT]) (REAL Alliance Expert opinion).*

When technically feasible and appropriate, SRS is preferred; however, there are times when WBRT is the preferred option. When it comes to the selection of local therapy and the sequencing of systemic therapy, MDT discussions are advised.

T-DM1 and T-DXd are valuable systemic agents in the treatment of HER2+ breast cancer. There are many clinical trials investigating the safety and efficacy of ADC in patients with BM.

Concurrent ADC and SRS is not recommended due to increased risk of radiation necrosis. This precautionary principle should also be applied to WBRT. Lebrow et al. conducted a retrospective cohort study in patients with metastatic breast cancer who received at least one course of SRS for BM and at least one dose of T-DM1, T-DXd, or sacituzumab govitecan (also an ADC used in metastatic breast cancer). SRS was considered concurrent with the ADC if administered fewer than 21 days after the last dose of ADC or fewer than 7 days before the next dose of ADC. The duration of 21 days was selected, as that is the time needed for the drug to be mostly removed from the body (i.e., approximately four half-lives [see Table 6]). The study concluded that concurrent ADC was associated with 4-fold greater higher risk of radionecrosis. The risk was greatest for larger tumours or for previously irradiated tumours [82].

**Recommendation** **21.** For patients with HER2+ metastatic breast cancer with parenchymal CNS disease, the decision to offer systemic therapy prior to local therapies should be individualized for each patient and ideally discussed at multidisciplinary* rounds. Key considerations include tumour burden and clinical symptoms. A multidisciplinary approach should be conducted to confirm if and when systemic therapy should be held during local CNS therapy to reduce the risk of toxicities and radiation necrosis (REAL Alliance Expert opinion). *Includes radiologist (neuroradiologist, if available), radiation oncologist, neurosurgeon, medical oncologist, and supportive care.

This recommendation pertains to the timing of systemic and local therapy. MDT discussions should take into consideration the tumour burden and the patient’s symptoms when making recommendations on sequencing of local and systemic therapies.

**Recommendation** **22.** *For patients with HER2+ metastatic breast cancer with active or progressive systemic disease in the presence of treated BM, the standard of care in the first line setting is trastuzumab + pertuzumab + taxane (strong recommendation).*

This recommendation refers to patients who are being treated in the first-line metastatic setting (i.e., de novo or relapse ≥6 months after completion of [neo]adjuvant therapy) and who have treated and thus stable BM. Treated BM does not alter the systemic treatment decision. Therefore, the treatment of these patients would be the same as Recommendation 11.

The standard-of-care first-line treatment in the metastatic setting for patients with HER2+ breast cancer is trastuzumab + pertuzumab + taxane as per the CLEOPATRA trial (see Recommendation 11). The CLEOPATRA trial excluded patients with CNS metastases. However, computed tomography (CT) or MRI of the brain was mandatory within 29 days of randomization in patients with clinical suspicion of BM [85].

Exploratory analyses were conducted on the development of CNS metastases in the CLEOPATRA trial [85]. The median time to CNS metastases and median OS were determined for patients who developed CNS metastases as the first site of disease progression. A similar proportion of patients in the placebo (51 of 406; 12.6%) and pertuzumab (55 of 402; 13.7%) arms developed CNS metastases as the first site of disease progression. However, the median time to progression favoured the pertuzumab arm, as did the OS, though the latter was not statistically significant.

Furthermore, the phase IIIb, single-arm PERUSE trial investigated first-line pertuzumab + trastuzumab + taxane therapy in HER2+ locally recurrent or metastatic breast cancer and included patients with CNS metastases if stable for ≥3 months before screening after receiving local therapy without HER2-targeted therapy [86]. In the overall population, median PFS was 20.7 months (95% CI 18.9–23.1) after a median follow-up of 68.7 months [46].

**Recommendation** **23.** *For patients with HER2+ metastatic breast cancer with stable BM whose disease has progressed on first line therapy, the standard of care options include T-DXd (preferred) and tucatinib + capecitabine + trastuzumab (strong recommendation).*

DESTINY-Breast03 investigated PFS and OS in patients with HER2+ unresectable or metastatic breast cancer where the disease had progressed during or after treatment with trastuzumab and taxane [49]. Patients were eligible if they had previously treated BM that was no longer symptomatic. At baseline, 43 patients (16%) and 39 patients (15%) had baseline CNS metastases in the T-DXd and T-DM1 groups, respectively. The primary endpoint of median PFS was 28.8 months with T-DXd versus 6.8 months with T-DM1 (HR 0.33, 95% CI 0.26–0.43; *p* < 0.0001). Median OS was not reached, with 72 (28%) overall survival events in the T-DXd group versus not reached with 97 (37%) overall survival events in the T-DM1 group [49]. A consistent OS benefit was observed across subgroups, including those with or without baseline BM [49].

The findings of DESTINY-Breast03 are extended by the pooled analysis of DESTINY-Breast01, 02, and 03 in patients with BM. Patients were categorized as treated/stable or untreated/active. Of the patients with BM who received T-DXd, 104 (70.3%) had treated/stable and 44 (29.7%) had untreated/active BM. The intracranial overall response rates (CR + PR in brain) in the T-DXd group were 45.2% and 45.4% for treated/stable and untreated/active BM, respectively. In the comparator group, the rates were 27.6% and 12.0% for treated/stable and untreated/active BM, respectively.

DESTINY-Breast12 confirmed a meaningful and durable intracranial response and provided further evidence supporting the use of T-DXd in patients with both stable and active BM [87]. DESTINY-Breast12 was a non-comparative study designed to evaluate the safety and efficacy of T-DXd in patients with HER2+ metastatic breast cancer in patients with and without baseline BM. In all, 504 patients were treated: 263 with baseline BM, and 241 without. Of the patients with baseline BM, 157 had previously treated and stable BM, and 106 had active BM (39 with untreated and 67 with previously treated and progressive). The 12-month PFS was 62.9% in patients with stable BM and 59.6% in patients with active BM. The CNS objective response rate was 79.2% in stable and 62.3% in active BM. The 12-month OS was similar in both patients with and without BM at baseline (~90%) [87].

HER2CLIMB is discussed in detail in Recommendation 23. This study investigated the efficacy and safety of tucatinib + capecitabine + trastuzumab in patients with and without BM. It is notable that HER2CLIMB allowed both active and stable brain metastases.

For patients with active BM and leptomeningeal carcinomatosis (LMC), there are limited treatment options. A real-world study, ROSET-BM, enrolled 104 patients with HER2+ breast cancer who had BM, which included 17 patients with both BM and LMC and 2 patients with LMC without BM. Patients were treated with T-DXd. The 12-month PFS and OS rates among patients with LMC were 60.7% (95% CI, 34.5–79.1%) and 87.1% (96% CI, 57.3–96.6%), respectively [88]. Also, case reports have documented the activity of tucatinib (in combination with capecitabine) in leptomeningeal disease [89].

**Recommendation** **24.** *For patients with HER2+ metastatic breast cancer and asymptomatic active (i.e., untreated) or stable brain metastases where local therapy is not indicated and whose disease has progressed during first-line therapy, options include tucatinib + capecitabine + trastuzumab or T-DXd. Such cases should be reviewed by the MDT to determine sequencing of local and systemic therapies (strong recommendation).*

The HER2CLIMB study enrolled patients who had advanced HER2+ breast cancer who had been previously treated with trastuzumab, pertuzumab, and T-DM1 [53]. Unlike historical clinical trials that excluded active BM, patients with stable (treated and not progressing) and active (untreated asymptomatic or treated and progressing) BM were eligible unless they needed immediate local intervention. Patients were randomized to either tucatinib or a placebo in combination with capecitabine and trastuzumab. The PFS at 1 year in patients with BM was 24.9% in the tucatinib-combination group and 0% in the placebo-combination group (HR 0.48, 95% CI 0.34–0.69; *p* < 0.001) [53]. Further analyses of the intracranial efficacy found that among the 174 patients with active BM, the 1-year CNS-PFS was 35% in the tucatinib arm compared to 0% in the control arm. Furthermore, the risk of progression or death was reduced by 64% in the tucatinib group (HR 0.36, 95% CI 022–0.57; *p* < 0.0001). For patients with stable BM, the estimated 1-year CNS-PFS was 53.3% and 0% in the tucatinib and control arms, respectively. Risk of progression or death was reduced by 69% in the tucatinib arm (HR 0.31, 95% CI 0.14–0.67; *p* = 0.002) [90].

As described in Recommendation 22, the results of DESTINY-Breast12 provide further evidence in support of T-DXd in patients with either stable or active BM [87].

Based on this evidence, ASCO recommends tucatinib in combination with capecitabine and trastuzumab in patients with HER2+ metastatic breast cancer who have asymptomatic BM and whose disease has progressed during at least one HER2-directed therapy in the metastatic setting.

The REAL Alliance endorses this recommendation for Canadian practice and adds that T-DXd is also a treatment option for patients with stable or active BM based on DESTINY-Breast03 and DESTINY-Breast12.

**Recommendation** **25.** *For patients with HER2+ metastatic breast cancer and active brain metastases whose disease has progressed on second-line systemic therapy, the standard of care is tucatinib + capecitabine + trastuzumab (preferred if not used in second line) or T-DXd (preferred if not used in second line or if there are signs of extensive systemic disease) (strong recommendation).*

The REAL Alliance recommends selecting treatments based on regimens that have not yet been utilized, disease biology, toxicities, patient preferences, and quality of life considerations.

All REAL Alliance recommendations for BM are outlined in an algorithm (see Figure 2).

### 3.4. Special Considerations

The REAL Alliance recognizes two situations that are often encountered in clinical practice, namely drug holidays and surgery to the primary site in patients with metastatic disease (see Table 7).

**Recommendation** **26.** *In patients with HER2+ metastatic breast cancer, a drug holiday can be considered in cases where there is stable disease on imaging (REAL Alliance Expert opinion).*

The REAL Alliance defines a drug holiday as a break from treatment of varying lengths, depending on the situation, with the appropriate clinical monitoring (for new symptoms or disease progression) and patient support. The REAL Alliance provides this expert opinion on drug holidays as they represent a common situation in practice, yet there is little evidence to guide decision-making. Patients with stable disease on imaging for whom a drug holiday is being considered should ideally be asymptomatic from their disease. Another situation where a drug holiday may be considered is in patients who are experiencing cumulative toxicities in the presence of stable disease on imaging. If a drug holiday is implemented, MDT discussions should take place on when to end the drug holiday and what to do if disease progression occurs during the drug holiday.

**Recommendation** **27.** *In patients with HER2+ de novo metastatic breast cancer who have a clinical complete response in the metastatic sites from chemotherapy + HER2-directed therapy and whose PET scan is negative, but who have breast-only residual disease, surgery can be considered at the site of the primary tumour to obtain no evidence of disease (NED) (REAL Alliance Expert opinion).*

The situation arises in practice where surgery to the primary tumour is considered in patients with metastatic disease who have experienced a CR in the metastatic sites. Clinicians will be required to decide on the best treatment approach to control the primary site, as currently there is little evidence to guide this decision. MDT discussions, along with patient preferences, are key in treatment decision-making.

A retrospective cohort review using the National Cancer Database (NCDB) investigated women with HER2+ stage IV breast cancer, comparing those who did and did not undergo definitive breast surgery [91]. The final study sample included 3231 patients, of whom 1130 (35%) underwent surgery on the primary site and 2889 (89.4%) received chemotherapy + HER-2-directed therapy. The overall mortality hazard was significantly decreased in patients who received chemotherapy/targeted therapy (HR 0.76, *p* = 0.008), endocrine therapy (HR 0.70, *p* = 0.0006), and surgery (HR 0.52, *p* < 0.0001). The conclusion of this study was that surgery of the primary site for metastatic HER2+ breast cancer is associated with improved survival in selected patients (HR 0.56, 95% CI 0.40–0.77) [91].

Previous studies have provided mixed results. A retrospective analysis reported by Lu et al. evaluated 96 patients with de novo stage IV HER2+ breast cancer, including 24 (25%) patients undergoing surgery at the primary site [90,92]. Patients in the surgical group had a longer median PFS (25.7 vs. 15.9 months) and median OS (79.1 vs. 48 months); however, it did not reach statistical significance. The authors concluded that primary site surgery does not improve the prognosis of de novo stage IV HER2-positive breast cancer [92].

Other studies in this area include the MF07-01 study conducted in Turkey, which concluded that survival rates were better in patients who received localized treatment compared to those who did not (10-year survival 19% vs. 5%) [93]. In contrast, the TATA study and the TBCRC013 study concluded that primary site surgery does not improve survival in stage IV breast cancer [94,95].

The patients described in this recommendation are very specific and perhaps not reflected in these studies.

## 4. Conclusions

These recommendations have been developed by a national group of Canadian breast oncologists, in collaboration with a patient organization, using the most current evidence. Like other international guidelines, these recommendations will be updated as new data become available. The REAL Alliance recommendations align with all other international guidelines, with the exception that the REAL Alliance recommends screening patients for asymptomatic brain metastases at the time of diagnosis with stage 4 HER2+ disease. The goal of the REAL Alliance is to promote best practices as well as timely and equitable access to treatments and care for patients with HER2+ breast cancer across Canada.

## Figures and Tables

**Table 1:** Summary of REAL Alliance recommendations for the treatment of HER2+ early breast cancer and comparison with those from ESMO and ASCO.

Recommendations for Early Breast Cancer		REAL	ESMO	ASCO
**1**	*cT1a and b, cN0* **For patients with HER2+ early breast cancer cT1a and b (i.e., ≤1 cm) without evidence of nodal disease (cN0)**, the standard of care is timely surgery followed by adjuvant treatment depending on the pathologic staging of disease (*see Recommendations 3–5)*.	Strong recommendation ●●	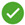 cT1 cN0	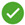
**2**	*cT1c, cN0* **For patients with HER2+ early breast cancer cT1c (i.e., >1 to ≤2 cm) without evidence of nodal disease (cN0)**, the standard of care is surgery followed by adjuvant treatment. However, due to current global practices, consideration can be given to neoadjuvant treatment followed by surgery and adjuvant treatment.	Strong recommendation ●● Strong consideration ○○	NC No specific guideline for cT1c	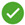 For high-risk node-negative disease, pertuzumab may be used with trastuzumab
**3**	*pT1, pN0* **For patients with HER2+ early breast cancer with pT1 without evidence of nodal disease (pN0)**, the standard of care adjuvant systemic treatment is paclitaxel + trastuzumab for 12 weeks followed by trastuzumab monotherapy for 9 months.	Strong recommendation ●●	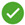	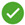
**4**	*≥pT2, pN0* **Although neoadjuvant treatment is preferred, for those patients who are treated with upfront surgery and are *then* found to have ≥pT2 pN0 disease**, the standard of care is adjuvant chemotherapy + trastuzumab.	Strong recommendation ●●	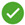	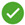
**5**	*≥cT2 or cN+* **For patients with HER2+ early breast cancer with ≥cT2 or those with nodal disease (cN+)**, the standard of care is neoadjuvant therapy with trastuzumab + pertuzumab + chemotherapy.	Strong recommendation ●●	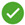	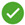
**6**	*pN+* **Although neoadjuvant treatment is preferred, for those patients who are treated with upfront surgery and are *then* found to have nodal disease in the pathological specimen (pN+)**, the standard of care is adjuvant chemotherapy + trastuzumab with consideration given to the addition of pertuzumab.	Strong consideration ○○	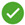 Chemo + HP followed by HP to complete the year	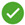
**7**	*Pathologic complete response* **For patients with HER2+ early breast cancer in whom a pathologic complete response is determined in the surgical specimen after completion of neoadjuvant trastuzumab + pertuzumab + chemotherapy**, the standard of care is trastuzumab for a total of 1 year.	Strong recommendation ●●	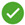 If cN0 at initial diagnosis If cN+ at initial diagnosis, then HP	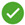
**8**	*Residual invasive disease* **For patients with HER2+ early breast cancer in whom residual invasive disease is detected pathologically in the surgical specimen of the breast or axillary lymph nodes after completion of neoadjuvant trastuzumab + pertuzumab + chemotherapy**, the standard of care is to treat with trastuzumab emtansine (T-DM1) for 14 cycles in the adjuvant setting.	Strong recommendation ●●	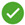	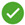
**9**	*Hormone receptor-positive disease* **Although there is not a survival benefit, for patients with HER2+ HR+ and N+ disease who have completed (neo)adjuvant chemotherapy + trastuzumab**, extended adjuvant treatment with neratinib for 1 year after completion of trastuzumab-based adjuvant therapy can be considered to decrease recurrence.	Moderate recommendation ●	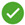	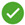

○, REAL Alliance expert opinion; ●, moderate recommendation; ○○, strong consideration; ●●, strong recommendation. 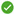, alignment; NC, not covered. Pink text describes the tumour and nodal status, and bolded text describes the patient. ASCO, American Society of Medical Oncology; ESMO, European Society of Medical Oncology; HP, trastuzumab + pertuzumab; HR, hormone receptor.

**Table 3:** Summary of REAL Alliance recommendations for the treatment of HER2+ metastatic breast cancer and comparison with those from ESMO and ASCO.

Recommendations for Metastatic Breast Cancer		Strength of Recommendation	ESMO	ASCO
**10**	*Repeat biopsy* When safe and feasible, repeat biopsy should be performed in **all patients whose disease relapses on or after adjuvant treatment**.	Strong recommendation ●●	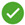	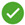
**11**	*1L treatment late relapse* **For patients with de novo HER2+ (HR±) metastatic breast cancer who have not received prior HER2-directed therapy or chemotherapy for metastatic disease or with disease that relapses >6 months after completion of (neo)adjuvant chemotherapy + HER2-directed therapy**, the standard of care is trastuzumab + pertuzumab + taxane chemotherapy followed by trastuzumab + pertuzumab +/− ET maintenance therapy.	Strong recommendation ●●	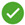	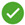
**12**	*1L treatment early relapse* **For patients with HER2-positive (HR±) metastatic breast cancer whose disease relapses ≤6 months after completion of (neo)adjuvant chemotherapy + HER2-directed therapy**, the recommended treatment is as per the second-line recommendation (*see Recommendation 13*).	Strong recommendation ●●	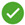 (relapse ≤ 12 mos)	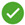
**13**	*2L treatment* **For patients with HER2-positive metastatic breast cancer whose disease has progressed on first-line HER2-directed therapy**, the standard of care is trastuzumab deruxtecan (T-DXd) in the absence of contraindications.	Strong recommendation ●●	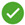	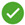
**14**	*3L treatment* **For patients with HER2+ metastatic breast cancer whose disease has progressed after at least 2 HER2-directed therapies** the recommendation for treatment is tucatinib + capecitabine + trastuzumab (can be considered earlier if brain metastasis is present).	Moderate recommendation ●	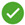	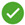
**15**	*4L and later treatment* **For patients with HER2+ metastatic breast cancer whose disease has progressed after at least 3 HER2-directed therapies**, the recommendation based on evidence is to continue HER2-directed therapy. Options include T-DXd (preferred option, if not previously used); chemotherapy + trastuzumab or another monoclonal antibody; trastuzumab emtansine (T-DM1); neratinib +/- capecitabine; and lapatinib + capecitabine.	Moderate recommendation ●	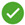	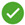

○, REAL Alliance expert opinion; ●, moderate recommendation; ○○, strong consideration; ●●, strong recommendation. 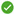, alignment; NC, not covered. Pink text describes the tumour and nodal status, and bolded text describes the patient. 1L, first-line; 2L, second-line; 3L, third-line; 4L, fourth-line; ASCO, American Society of Medical Oncology; ESMO, European Society of Medical Oncology.

**Table 5:** Summary of REAL Alliance recommendations for the treatment of HER2+ metastatic breast cancer with brain metastases and comparison with those from ESMO and ASCO.

Recommendations for Brain Metastases		Strength of Recommendation	ESMO	ASCO
**16**	*CNS screening* **For patients with HER2+ metastatic breast cancer who have symptoms suggestive of CNS metastases**, appropriate diagnostic investigations for CNS metastases are essential.	Strong recommendation ●● REAL Alliance expert opinion ○	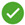 If detection of CNS metastases will alter the choice of systemic therapy	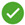 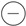
	**For patients with HER2+ metastatic breast cancer**, screening for CNS metastases should be considered in **asymptomatic patients** at baseline in the metastatic setting and at disease progression.			
**17**	*Multidisciplinary care* **For patients with a history of HER2+ metastatic breast cancer who are diagnosed with brain metastases**, multidisciplinary care with representation from radiology, radiation oncology, neurosurgery, medical oncology, and supportive care is the standard of care, with the multidisciplinary team providing recommendations on sequencing of local and systemic therapies.	REAL Alliance expert opinion ○	NC	NC
**18**	*Characteristics of CNS disease at screening* **For patients with a history of HER2+ metastatic breast cancer but without other extracranial systemic disease who present with oligometastatic brain disease amenable to local therapy**, there is insufficient evidence to make a recommendation for a change in systemic therapy. Multidisciplinary care is the standard of care, and the multidisciplinary team is to make recommendations on sequencing of local and systemic therapies in such patients.	REAL Alliance expert opinion ○	NC	NC
**19**	*Characteristics of CNS disease at screening* **For patients with a history of HER2+ metastatic disease who present with asymptomatic, low volume, newly diagnosed brain metastases**, treatment should be discussed by MDT incorporating patient values with treatment options including initial HER2-directed systemic therapy versus upfront local therapy.	REAL Alliance expert opinion ○	NC	NC
**20**	*Characteristics of CNS disease at screening* **For patients with a history of HER2+ metastatic disease who present with symptomatic, newly diagnosed brain metastases**, upfront stereotactic radiosurgery is a reasonable approach when technically feasible (and often preferred over whole brain radiotherapy).	REAL Alliance expert opinion ○	NC	NC
**21**	*Characteristics of CNS disease at screening* **For patients with HER2+ metastatic breast cancer with parenchymal CNS disease**, the decision to offer systemic therapy prior to local therapies should be individualized for each patient and ideally discussed at multi-disciplinary rounds. Key considerations include tumour burden and clinical symptoms. A multidisciplinary approach should be conducted to confirm if and when systemic therapy should be held during local CNS therapy to reduce the risk of toxicities and tumour necrosis.	REAL Alliance expert opinion ○	NC	NC
**22**	*1L treatment stable brain metastases* **For patients with HER+ metastatic breast cancer with active or progressive systemic disease in the presence of treated brain metastases**, the standard of care in the first line setting is trastuzumab + pertuzumab + taxane.	Strong recommendation ●●	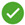	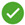
**23**	*2L treatment stable brain metastases* **For patients with HER2+ metastatic breast cancer with stable brain metastases whose disease has progressed on first line therapy**, the standard of care options are trastuzumab deruxtecan (T-DXd [preferred]), or tucatinib + capecitabine + trastuzumab.	Strong recommendation ●●	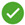	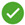
**24**	*2L treatment active brain metastases* **For patients with HER2+ metastatic breast cancer and asymptomatic active (i.e., untreated) or stable brain metastases where local therapy is not indicated and whose disease has progressed on first-line therapy**, options include tucatinib + capecitabine + trastuzumab or T-DXd. Such cases should be reviewed by the multidisciplinary team to determine sequencing of local and systemic therapies.	Strong recommendation ●●	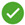	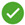
**25**	*3L treatment* **For patients with HER2+ metastatic breast cancer and active brain metastases whose disease has progressed on second-line systemic therapy**, the standard of care is tucatinib + capecitabine + trastuzumab (preferred if not used in second line) or T-DXd (preferred if not used in second line or if there are signs of extensive systemic disease)	Strong recommendation ●●	NC	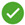

○, REAL Alliance expert opinion; ●, moderate recommendation; ○○, strong consideration; ●●, strong recommendation. 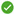, alignment; 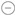, some variation; NC, not covered. Pink text describes the tumour and nodal status, and bolded text describes the patient. 1L, first-line; 2L, second-line; 3L, third-line; ASCO, American Society of Medical Oncology; ESMO, European Society of Medical Oncology.

**Table 6:** Half-life and guidance for interrupting and stopping treatment based on adverse reactions with antibody–drug conjugates.

Antibody–Drug Conjugate	Trastuzumab Deruxtecan [83]	Trastuzumab Emtansine [84]
Half life	5.6 days	3.1 to 4.5 days

**Table 7:** Summary of REAL Alliance recommendations for special situations and comparison with those from ESMO and ASCO.

Recommendations for Other Areas of Interest		Strength of Recommendation	ESMO	ASCO
**26**	*Drug holiday* **In patients with HER2+ metastatic breast cancer**, a drug holiday can be considered in cases where there is stable disease based on imaging.	REAL Alliance expert opinion ○	NC	NC
**27**	*Multidisciplinary care* **In patients with HER2+ de novo metastatic breast cancer**, who have a clinical complete response in the metastatic sites from chemotherapy + HER2-directed therapy and whose PET scan is negative, but who have breast-only residual disease, surgery can be considered at the site of the primary tumour to obtain no evidence of disease.	REAL Alliance expert opinion ○	NC	NC

○, REAL Alliance expert opinion; NC, not covered. Pink text describes the clinical situation, and bolded text describes the patient. ASCO, American Society of Medical Oncology; ESMO, European Society of Medical Oncology.

## Data Availability

No new data was generated.

## Supplementary Materials

The following supporting information can be downloaded at: https://www.mdpi.com/article/10.3390/curroncol31110484/s1, Figure S1: PRISMA Flow Diagram; Figure S2: Voting Results; Table S1: Evidence tables.
