# Supplementary material for: Guidance for Canadian Breast Cancer Practice: National Consensus Recommendations for the Systemic Treatment of Patients with HER2+ Breast Cancer in Both the Early and Metastatic Setting
Source: Curr Oncol. 2024 Oct 24;31(11):6536–67. doi: 10.3390/curroncol31110484 (PMC11593131; doi:10.3390/curroncol31110484)
Supplement: Supplementary file 1 [file curroncol-31-00484-s001.zip › curroncol-3245069-supplementary.pdf]

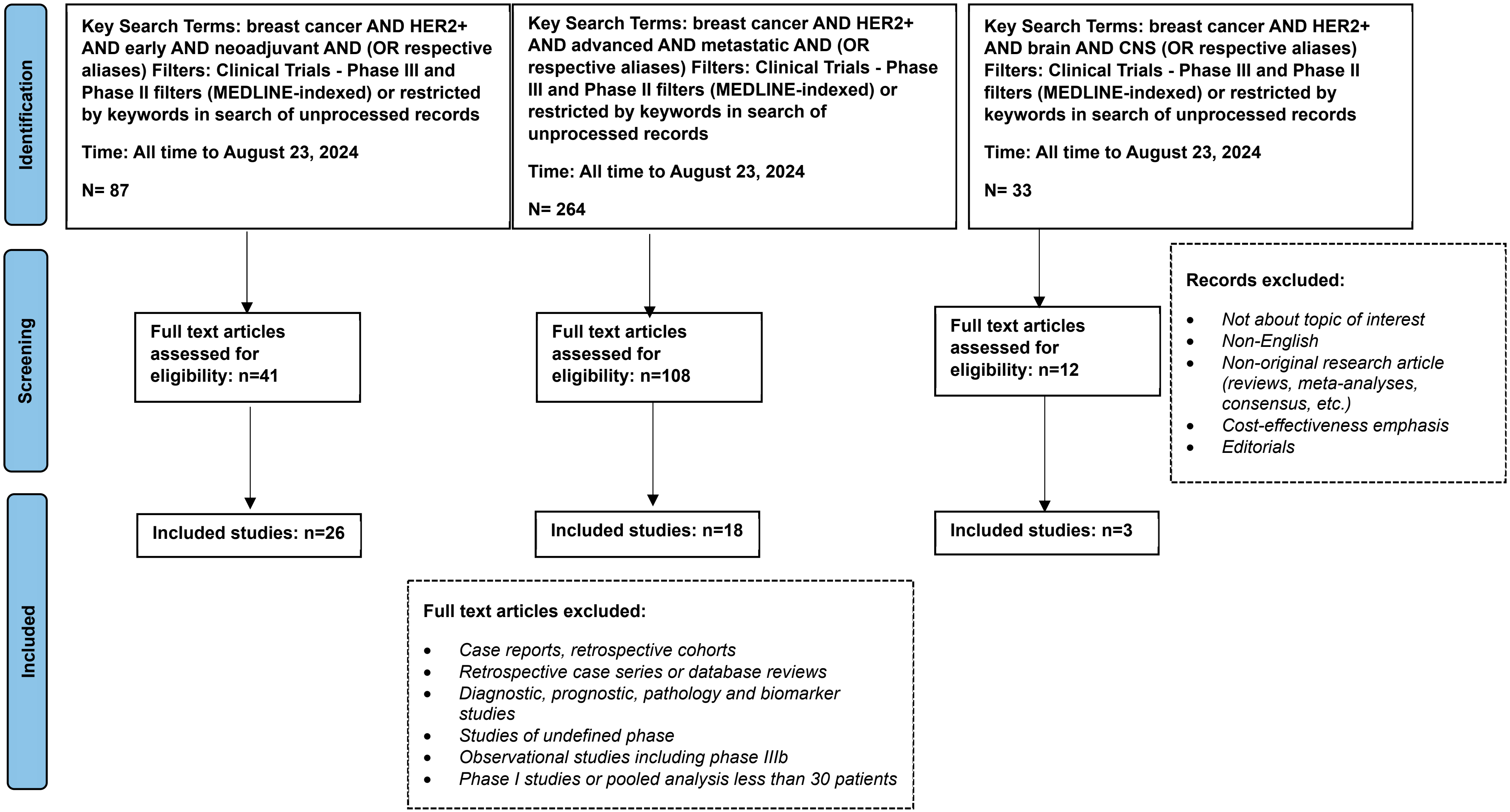

Supplement Figure S1. PRISMA Flow Diagram

Adapted from: Page MJ, McKenzie JE, Bossuyt PM, Boutron I, Hoffmann TC, Mulrow CD, et al. The PRISMA 2020 statement: an updated guideline for reporting systematic reviews. BMJ 2021;372:n71. For more information, visit http://www.prisma-statement.org/.

Supplemental Table S1. Characteristics of Included Studies Assessing early and advanced systemic therapies for HER2+ Breast Cancer.

Study	Treatment Line	Patient Population	Arms of Study	Number of Analyzed Patients by Arm	Median Follow-Up	Efficacy Outcome
Tolaney SM. et al. (2015)<br>APT<br>Phase III	Adjuvant	HER2+, ≤ 3cm	Paclitaxel + Trastuzumab for 12 weeks followed by 40 weeks of trastuzumab	406	48 months	3-year IDFS:<br>99%
Tolaney SM. et al. (2023)<br>APT<br>Phase III extended observation	Adjuvant	HER2+, ≤ 3cm	Paclitaxel + Trastuzumab for 12 weeks followed by 40 weeks of trastuzumab	406	130 months	10-year IDFS:<br>91%<br><br>10-year OS:<br>94%
Faleh, S. et al.<br>(2023)<br>Retrospective Study	Observational	HER2+, clinical stage I-II	NA	3333	NA	Pathological nodal metastasis:<br>- T1a 9%<br>- T1b 10%<br>- T1c 23%<br>- T2 37%
von Minckwitz G. et al.<br>(2019)<br><br>KATHERINE<br>Phase III	Adjuvant	HER2+ without pCR after neoadjuvant taxane (± anthracycline) and trastuzumab	1. T-DM1<br>2. Trastuzumab	1. 743<br>2. 743	41.4 months	3-year IDFS:<br>1. 88%<br>2. 77%
Mamounas EP. et al.<br>(2021)<br>KATHERINE<br>subgroup analysis	Adjuvant	HER2+ without pCR after neoadjuvant taxane (± anthracycline) and trastuzumab	1. T-DM1<br>2. Trastuzumab	1. 45<br>2. 32	41.4 months	3-year IDFS:<br>1. 100%<br>2. 81%
Tolaney SM. et al.<br>(2021)<br>ATEMPT	Adjuvant	HER2+, Stage I	1. T-DM1<br>2. Paclitaxel + trastuzumab	1. 383<br>2. 114	36 months	3-year IDFS:<br>1. 97.8%<br>2. 93.4%

Huang L. et al.<br>(2015)<br>ABX-1201<br>Phase II	Neo-adjuvant	HER2+, Stage II-III (except inflammatory)	1. Nab-paclitaxel<br>2. Paclitaxel + carboplatin	1. 30<br>2. 90	NA	pCR<br>1. 27%<br>2. 26%
Nakatsukasa K. et al.<br>(2017)<br>prospective multicenter study	Neo-adjuvant	HER2+, > 1 cm but <7 cm and a tumor stage of N0-1	Docetaxel<br>+ cyclophosphamide<br>+ trastuzumab	41	NA	pCR:<br>44%
Cameron D. et al.<br>(2017)<br>BIG-01-01 (HERA)<br>Phase-III long term follow-up	Adjuvant	HER2+, after completion of all primary therapy	1. 1-year Trastuzumab after chemotherapy<br>2. 2-year Trastuzumab after chemotherapy<br>3. Chemotherapy alone	1. 1702<br>2. 1700<br>3. 1697	132 months	11-year OS:<br>1. 79%<br>2. 80%<br>3. 73%<br>Estimated 10-year DFS:<br>1. 63%<br>2. 69%<br>3. 69%
Fehrenbacher L. et al.<br>(2019)<br>NSABP B-47/NRG<br>Phase III	Adjuvant	HER2+, high-risk primary	1. Trastuzumab + chemotherapy<br>2. Chemotherapy alone	1. 1598<br>2. 1602	46 months	5-year IDFS:<br>1. 92%<br>2. 92%
Miller KD. et al.<br>(2018)<br>E5103<br>Phase III	Adjuvant	HER2+, with risk of systemic recurrence and without prior chemotherapy	1. Chemotherapy<br>2. Bevacizumab + chemotherapy<br>3. Chemotherapy + extended bevacizumab	1. 1000<br>2. 1986<br>3. 2008	47.5 months	5-year IDFS:<br>1. 84%<br>2. 85%<br>3. 80%<br>5-year OS:<br>1. 90%<br>2. 86%<br>3. 90%
Gianni L. et al.<br>(2012)<br>NeoSphere<br>Phase II	Neoadjuvant	HER2+, operable (T2–3, N0–1, M0), locally advanced (T2–3, N2–3, M0 or T4a–c, any N, M0), or	1. Trastuzumab + docetaxel<br>2. Trastuzumab + pertuzumab + docetaxel	1. 107<br>2. 107<br>3. 107<br>4. 96	24 months	pCR:<br>1. 29.0%<br>2. 45.8%

		inflammatory (T4d, any N, M0)	3. Pertuzumab + docetaxel<br>4. Trastuzumab + pertuzumab			3. 16.8%<br>4. 24.0%
Gianni L. et al. (2016)<br>NeoSphere follow-up<br>Phase II	Neoadjuvant	HER2+, operable (T2–3, N0–1, M0), locally advanced (T2–3, N2–3, M0 or T4a–c, any N, M0), or infl ammatory (T4d, any N, M0)	1. Trastuzumab + docetaxel<br>2. Trastuzumab + pertuzumab + docetaxel<br>3. Pertuzumab + docetaxel<br>4. Trastuzumab + pertuzumab	1. 107<br>2. 107<br>3. 107<br>4. 96	60 months	5-year PFS:<br>1. 81%<br>2. 86%<br>3. 73%<br>4. 73%
Shao Z. et al. (2020)<br>PEONY<br>Phase III	Neoadjuvant	HER2+, early or locally advanced, > 2cm	1. Pertuzumab + trastuzumab + docetaxel<br>2. Placebo +trastuzumab + docetaxel	1. 219<br>2. 110	12 months	tpCR:<br>1. 39%<br>2. 22%
Huang L. (2024)<br>PEONY follow-up<br>Phase III	Neoadjuvant	HER2+, early or locally advanced (> 2cm)	1. Pertuzumab + trastuzumab + docetaxel<br>2. Placebo +trastuzumab + docetaxel	1. 219<br>2. 110	60 months	5-year OS:<br>1. 93%<br>2. 90%<br><br>5-year EFS:<br>1. 85%<br>2. 74%
Swain SM. (2018)<br>BERENICE<br>Phase II	Neoadjuvant	HER2+, untreated inflammatory or locally advanced (>2cm or >5mm with N+)	1. ddAC + paclitaxel + trastuzumab + pertuzumab<br>2. FEC + docetaxel + trastuzumab + pertuzumab	1. 199<br>2. 198	14.5 months	pCR:<br>1. 62%<br>2. 61%

Dang C. et al.<br>(2022)<br>BERENICE follow-up<br>Phase III	Neoadjuvant	HER2+, untreated inflammatory or locally advanced (>2cm or >5mm with N+)	1. ddAC + paclitaxel + trastuzumab + pertuzumab<br>2. FEC + docetaxel + trastuzumab + pertuzumab	1. 199<br>2. 198	64.5 months	5-year OS:<br>1. 96%<br>2. 94%<br>5-year EFS:<br>1. 91%<br>2. 89%
Panet F. et al.<br>(2023)<br>Retrospective	Neoadjvant	HER2+	1. Pertuzumab + trastuzumab<br>2. Trastuzumab	1. 39<br>2. 44	NA	pCR:<br>1. 62%<br>2. 27%
Schneeweiss A. et al.<br>(2017)<br>TRYPHAENA<br>Phase II	Neoadjuvant	HER2+, operable, locally advanced or inflammatory breast cancer (>2 cm)	1. Pertuzumab + trastuzumab + FEC + docetaxel<br>2. Pertuzumab + trastuzumab + docetaxel + carboplatin<br>3. Pertuzumab + trastuzumab + FEC + docetaxel (sequential)	1. 73<br>2. 75<br>3. 77	61 months	3-year DFS:<br>1. 87%<br>2. 88%<br>3. 90%<br>3-year PFS:<br>1. 89%<br>2. 89%<br>3. 87%<br>3-year OS:<br>1. 94%<br>2. 94%<br>3. 93%
Van Ramshorst MS. et al.<br>(2018)<br>TRAIN-2<br>Phase III	Neoadjuvant	HER2+, Stage II-III	1. FEC + paclitaxel + carboplatin<br>2. Paclitaxel + carboplatine	1. 220<br>2. 218	19 months	pCR:<br>1. 67%<br>2. 68%
van der Voort A, et al.<br>(2021)<br>TRAIN-2 follow-up<br>Phase III	Neoadjvant	HER2+, Stage II-III	1. FEC + paclitaxel + carboplatin<br>2. Paclitaxel + carboplatine	1. 220<br>2. 218	48.8 months	3-year EFS:<br>1. 93%<br>2. 94%<br><br>3-year OS:<br>1. 98%<br>2. 98%

von Minckwitz G, et al. (2017)<br>APHINITY<br>Phase III	Neoadjuvant	HER2+, node-positive or high-risk node-negative	1. Pertuzumab + trastuzumab + chemotherapy<br>2. Placebo + trastuzumab + chemotherapy	1. 2400<br>2. 2405	36 months	3-year IDFS:<br>1. 1%<br>2. 2%<br><br>Node-positive IDFS:<br>1. 92%<br>2. 90%<br><br>Node-negative IDFS:<br>1. 98%<br>2. 98%
Piccart M. et al. (2021)<br>APHINITY follow-up<br>Phase III	Neoadjuvant	HER2+, node-positive or high-risk node-negative	1. Pertuzumab + trastuzumab + chemotherapy<br>2. Placebo + trastuzumab + chemotherapy	1. 2400<br>2. 2405	74 months	6-year IDFS:<br>1. 91%<br>2. 88%<br><br>Node-positive IDFS:<br>1. 88%<br>2. 83%<br><br>Node-negative IDFS:<br>1. 95%<br>2. 95%<br><br>6-year OS:<br>1. 95%<br>2. 94%
Martin M, et al. (2017)<br>ExteNET<br>Phase III	Neoadjuvant	HER2+, Stage I-IIIC, post-trastuzumab therapy	1. Neratinib<br>2. Placebo	1. 1420<br>2. 1420	62.4 months	5-year IDFS:<br>1. 90.2%<br>2. 87.7%
Chan A, et al. (2021)<br>ExteNet follow-up<br>Phase III	Neoadjuvant	HER2+, Stage I-IIIC, post-trastuzumab therapy	1. Neratinib<br>2. Placebo	1. 1420<br>2. 1420	62.4 months	5-year IDFS:<br>HR+/≤ 1-year group: 5% absolute benefit

						HR+/>1-year group: 1% absolute benefit<br><br>OS improvement:<br>HR+/≤ 1-year: 2% absolute benefit at 8 years
Cameron D, et al.<br>(2017)<br>HERA<br>Phase III	Neoadjuvant	HER2+, early-stage	1. Trastuzumab for 1 year<br>2. Trastuzumab for 2 years<br>3. Observation	1. 1702<br>2. 1700<br>3. 1697	132 months	10-year DFS:<br>1. 69%<br>2. 69%<br>3. 63%
Swain SM, et al.<br>(2020)<br>CLEOPATRA<br>Phase III	Metastatic	HER2+, metastatic	1. Pertuzumab + trastuzumab + docetaxel<br>2. Placebo + trastuzumab + docetaxel	1. 402<br>2. 406	96 months	Median OS:<br>1. 57.1 months<br>2. 40.8 months<br><br>8-year OS:<br>1. 37%<br>2. 23%
Baselga J. et al.<br>(2012)<br>CLEOPATRA<br>Phase III	Metastatic	HER2+, metastatic	1. Pertuzumab + trastuzumab + docetaxel<br>2. Placebo + trastuzumab + docetaxel	1. 402<br>2. 406	19.3 months	Median PFS:<br>1. 18.5 months<br>2. 12.4 months
Miles D. et al.<br>(2021)<br>PERUSE<br>Phase IIIb	Metastatic	HER2+, locally recurrent/metastatic	Pertuzumab + trastuzumab + taxane (docetaxel, paclitaxel, or nab-paclitaxel)	1436	68.4 months	Median PFS:<br>20.7 months<br><br>Median OS:<br>65.3 months
Rimawi M. et al.<br>(2018)<br>PERTAIN<br>Phase II	Metastatic	HER2+, HR+ metastatic/locally advanced	1. Pertuzumab + trastuzumab + AI<br>2. Trastuzumab + AI	1. 129<br>2. 129	31 months	Median PFS:<br>1. 18.9 months<br>2. 15.8 months
Curigliano G. et al.<br>(2024)<br>DESTINY-BREAST 06	Metastatic	HER2-low or HER2-ultralow, HR+ metastatic	1. T-DXd<br>2. Physician's choice of chemotherapy	1. 436 (HER2-low: 359, HER2-ultralow: 76)	18.6 months	Median PFS:<br>1. 13.2 months (HER2-low) and 13.2 months (HER2-ultralow)

Phase III				2. 430 (HER2-low: 354, HER2-ultralow: 76)		2. 8.1 months (HER2-low) and 8.3 months (HER2-ultralow)<br><br>12-month OS rate:<br>1. 88% (HER2-low) and 84% (HER2-ultralow)<br>2. 82% and 79% (HER2-ultralow)<br><br>ORR:<br>1. 56.5% (HER2-low) and 61.8% (HER2-ultralow)<br>2. 32% (HER2-low) and 26.% (HER2-ultralow)
Hurvitz SA. Et al. (2023) DESTINY-BREAST 03 Phase III	Metastatic	HER2+ metastatic	1. T-DXd<br>2. T-DM1	1. 261<br>2. 263	28 months	Median PFS:<br>1. 28.8 months<br>2. 6.8 months<br><br>m OS not reached in either group
Cortes J. et al. (2022) DESTINY-BREAST 03 Phase III subgroup analysis	Metastatic	HER2+ metastatic	1. T-DXd<br>2. T-DM1	1. 193 (1 line of previous anti-HER2 therapy) and 66 (≥2 line of previous anti-HER2 therapy)<br><br>2. 197 (1 line of previous anti-HER2 therapy) and 63 (≥2 line of previous anti-HER2 therapy)	NA	mPFS:<br>1. NE (1 line of previous anti-HER2 therapy) and 22.2 months (≥2 lines of previous anti-HER2 therapy)<br>2. 8.0 months (1 line of previous anti-HER2 therapy) and 4.2 (≥2 lines of previous anti-HER2 therapy)
Cortes J. et al. (2024) DESTINY-BREAST-03 Phase III follow-up	Metastatic	HER2+ metastatic	1. T-DXd<br>2. T-DM1	1. 261<br>2. 263	41 months	PFS:<br>1. 29.0 months<br>2. 7.2 months<br><br>36-month PFS rate:

						1. 46%<br>2. 12%<br><br>OS:<br>1. 52.6 months<br>2. 42.7 months
Andre F. et al.<br>(2023)<br>DESTINY-BREAST-02<br>Phase III	Metastatic	HER2+ metastatic, refractory to T-DM1	1. T-DXd<br>2. Physician's choice	1. 406<br>2. 202	20 months	PFS:<br>1. 17.8 months<br>2. 6.9 months
Modi S. et al.<br>(2020)<br>DESTINY-BREAST-01<br>Phase II	Metastatic	HER2+ metastatic, refractory to T-DM1	T-DXd	184	11.1 months	ORR:<br>61%<br><br>Median Response Duration:<br>14.8 months<br><br>m PFS:<br>16.4 months
Krop IE. et al.<br>(2014)<br>TH3RESA<br>Phase III	Metastatic	HER2+ advanced, pretreated with ≥2 HER2-directed regimens	1. T-DM1<br>2. Physician's choice	1. 404<br>2. 198	7 months	PFS:<br>1. 6.2 months<br>2. 3.3 months<br><br>OS (interim):<br>Trend favoring T-DM1 (HR 0.552, 95% CI 0.369-0.826, p=0.0034)
Krop IE. Et al.<br>(2017)<br>TH3RESA<br>Phase III follow-up	Metastatic	HER2+ advanced, pretreated with ≥2 HER2-directed regimens	1. T-DM1<br>2. Physician's choice	1. 404<br>2. 198	31 months	OS:<br>1. 22.7 months<br>2. 15.8 months
Verma S. et al.<br>(2012)<br>EMILIA<br>Phase III	Metastatic	HER2+ advanced, previously treated with trastuzumab and a taxane	1. T-DM1<br>2. Lapatinib + capecitabine	1. 495<br>2. 496	8 months	PFS:<br>1. 9.6 months<br>2. 6.4 months<br><br>OS:

						1. 30.9 months<br>2. 25.1 months<br><br>Objective Response Rate:<br>1. 43.6%<br>2. 30.8%
Powell CA. Et al.<br>(2022)<br>Pooled analysis	NA	Patients treated with T-DXd across multiple cancers (breast, gastric, lung, colorectal, other)	T-DXd	1150	range:<br>0.7-56.3 months	Incidence of Drug-Related ILD/Pneumonitis:<br>15.4% (Grade 5: 2.2%)<br>Most cases were low grade (Grade 1 or 2): 77.4%
Murthy RK. et al.<br>(2019)<br>HER2CLIMB<br>Phase III	Metastatic later lines (Brain metastasis)	HER2+ metastatic with prior treatment including trastuzumab, pertuzumab, and TDM1	1. Tucatinib + trastuzumab + capecitabine<br>2. Placebo + trastuzumab + capecitabine	1. 404<br>2. 198	14 months	mPFS:<br>1. 7.8 months<br>2. 5.6 months<br><br>mOS:<br>1. 21.9 months<br>2. 17.4 months<br><br>PFS in Brain Metastases:<br>1-year PFS:<br>1. 25%<br>2. 0%<br><br>mPFS:<br>1. 7.6 months<br>2. 5.4 months
Saura C. et al.<br>(2024)<br>DESTINY-BREAST-01<br>Phase II	Metastatic	HER2+ metastatic mBC previously treated with T-DM1	T-DXd	184	27 months	ORR:<br>62.0%<br><br>mOS:<br>29.1 months<br><br>mPFS:

						19.4 months<br><br>mDoR:<br>18.2 months
Tolaney SM. Et al.<br>(2020)<br>monarcHER<br>Phase II	Metastatic	HER2+ advanced with unresectable, locally advanced, recurrent or metastatic	1. Abemaciclib + trastuzumab + fulvestrant<br>2. Abemaciclib + trastuzumab<br>3. Standard-of-care chemotherapy + trastuzumab	1. 79<br>2. 79<br>3. 79	19 months	mPFS:<br>1. 8.3 months<br>2. 5.7 months<br>3. 5.7 months
Suara C. et al.<br>(2020)<br>NALA<br>Phase III	Metastatic	HER2+ metastatic with ≥ 2 previous HER2-directed regimens	1. Neratinib + capecitabine<br>2. Lapatinib + capecitabine	1. 307<br>2. 314<br>Note: 16% with CNS involvement	30 months	mPFS:<br>1. 5.6 months<br>2. 5.5 months<br><br>mOS:<br>1. 21.0 months<br>2. 18.7 months
Gueyer CE. Et al.<br>(2006)<br>NCT00086814<br>Phase III	Metastatic	HER2+, locally advanced or metastatic that had progressed after treatment including anthracycline, taxane, and trastuzumab	1. Lapatinib + capecitabine<br>2. Capecitabine	1. 163<br>2. 161		mPFS:<br>1. 8.4 months<br>2. 4.4 months<br><br>mTTP<br>1. 8.4 months<br>2. 4.1 months
Rugo HS. Et al.<br>(2021)<br>SOPHIA<br>Phase III	Metastatic	HER2+ metastatic previously treated with HER2-targeted therapies	1. Margetuximab + chemotherapy<br>2. Trastuzumab + chemotherapy	1. 266<br>2. 270	NA	Risk reduction:<br>24% relative risk reduction in hazard of progression<br><br>mOS:<br>1. 21.6 months<br>2. 19.8 months

Gabos Z. et al.<br>(2006)<br>Prognostic study	Observational<br>(brain metastasis)	Newly diagnosed HER2+ and HER2-	1. HER2+<br>2. HER2-	1. 301<br>2. 363	46.8 months	Brain metastasis incidence:<br>1. 9%<br>2. 2%
Darlix A. et al.<br>(2019)<br>Retrospective study	Observational<br>(CNS metastasis)	HER2-/HR+, HER2+/HR+, HER2+/HR-, TNBC metastatic	1. HER2-/HR+<br>2. HER2+/HR+<br>3. HER2+/HR-<br>4. TNBC	1. 8654<br>2. 1480<br>3. 1010<br>4. 2354	42.8 months	mOS after CNS metastasis:<br>1. 7.1 months<br>2. 18.9 months<br>3. 13.1 months<br>4. 4.4 months
Kuksis M. et al.<br>(2021)<br>Meta-analysis	Observational<br>(brain metastasis)	HER2+, TNBC, HR+/HER2-, metastatic	1. HER2+<br>2. TNBC<br>3. HR+/HER2-	1. 17 studies for HER2+<br>2. 6 studies for TNBC<br>3. 4 studies for HR+/HER2-	1. 30.7 months<br>2. 32.8 months<br>3. 33.0 months	Pooled cumulative incidence of CNS metastesis:<br>1. 31%<br>2. 32%<br>3. 15%
Wang XY. Et al.<br>(2022)<br>Population-based Cohort Study	Observational<br>(brain metastasis)	HER2-/HR+, HER2+/HR+, HER2+/HR-, TNBC metastatic	1. HR+ /HER2-<br>2. HER2+/HR+<br>3. HER2+/HR-<br>4. TNBC<br>5. Unknown	1. 1215<br>2. 310<br>3. 200<br>4. 258<br>5. 1933	19.3 months	Cumulative incidence of brain metastases:<br>1. 12.1%<br>2. 28.1%<br>3. 34.7%<br>4. 21.9%<br><br>Median time from diagnosis to brain radiotherapy:<br>1. 19.8 months<br>2. 16.8 months<br>3. 15.1 months<br>4. 7.5 months
Loibl S. et al.<br>(2024)<br>KATHERINE<br>Phase III followup	Adjuvant	HER2+ early with residual invasive disease post-neoadjuvant chemotherapy	1. T-DM1<br>2. Trastuzumab	1. 740<br>2. 720	101 months	7-year IDFS:<br>1. 81%<br>2. 67%<br><br>7-year OS:<br>1. 89%<br>2. 84%

Brufsky AM. Et al. (2011) registHER prospective study	Observational (CNS metastasis)	HER2+ metastatic	1. CNS metastases<br>2. No CNS metastases	1. 377<br>2. 635	N/A	mOS after CNS diagnosis:<br>1. Trastuzumab: 17.5 months vs. no trastuzumab: 3.8 months<br>2. Chemotherapy: 16.4 months vs. no chemotherapy: 3.7 months<br>3. Surgery: 20.3 months vs. no surgery: 11.3 months<br>4. Radiotherapy: 13.9 months vs. no radiotherapy: 8.4 months (P = 0.134)<br>HR for chemotherapy: 0.64, P = 0.002
Pasquier D. et al. (2020) ESME MBC Cohort Study	Observational (CNS metastasis)	HER2+, HR+, and TNBC metastatic	1. Patients with CNS metastases	16,701 patients in total; 24.6% developed CNS metastases	42.8 months	mOS:<br>7.9 months<br><br>mNPFS:<br>5.5 months<br><br>Factors affecting NPFS:<br>- Age >70 years (HR = 1.40; 95% CI: 1.24-1.57)<br>- Triple-negative tumors (HR = 1.87; 95% CI: 1.71-2.06)<br>- HER2+/HR- tumors (HR = 1.14; 95% CI: 1.02-1.27)<br>- ≥3 metastatic sites (HR = 1.32; 95% CI: 1.21-1.43)<br>- ≥3 previous treatment lines (HR = 1.75; 95% CI: 1.56-1.96)
Kim GM. Et al. (2024) Prospective cohort study	Observational (brain metastasis)	HER2+ and TNBC metastatic	1. TNBC<br>2. HER2<br>3. Luminal-HER2	1. 75<br>2. 43<br>3. 29	20.1 months	Cumulative incidence of asymptomatic brain metastasis:<br>1. 24%

						2. 14%<br>3. 21%<br><br>mOS after brain metastasis diagnosis:<br>23.7 months for asymptomatic vs 7.3 months for symptomatic brain metastasis
Soon YY. et al. (2014)<br>Meta-analysis of RCTs	Surgery<br>(brain metastasis)	Patients with 1-4 brain metastases from systemic cancer	1. Surgery/SRS + WBRT<br>2. Surgery/SRS alone	5 RCTs, 663 patients total	N/A	Adding WBRT decreased the relative risk of intracranial disease progression at 1 year by 53% (RR 0.47, 95% CI 0.34-0.66, P < 0.0001).<br><br>No clear evidence of OS benefit (HR 1.11, 95% CI 0.83-1.48, P = 0.47) or PFS benefit (HR 0.76, 95% CI 0.53-1.10, P = 0.14).<br><br>High risk of bias impacting NF, HRQL, and neurological adverse events assessment.
Swain SM. et al. (2014)<br>CLEOPATRA Phase III exploratory	Metastatic<br>(brain metastasis)	HER2+ metastatic	1. Pertuzumab + trastuzumab + docetaxel<br>2. Placebo + trastuzumab + docetaxel	1. 402<br>2. 406	N/A	Incidence of CNS metastases as first site of progression:<br>1. 14%<br>2. 13%<br><br>Median time to CNS metastases:<br>1. 15.0 months<br>2. 11.9 months<br><br>mOS:<br>1. 34.4 months<br>2. 26.3 months

						No statistically significant difference in survival curves for log-rank test (P = 0.1139), significant for Wilcoxon test (P = 0.0449).
Lin NU. et al. (2020)<br>HER2CLIMB<br>Phase II<br>Exploratory analysis	Metastatic<br>(brain metastasis)	HER2+ metastatic with prior treatment including trastuzumab, pertuzumab, and TDM1 and with brain metastasis	1. Tucatinib + Trastuzumab + Capecitabine<br>2. Placebo + Trastuzumab + Capecitabine	1. 404<br>2. 198	N/A	Median intracranial progression-free survival (CNS-PFS):<br>1. 9.9 months<br>2. 4.2 months<br><br>m OS:<br>1. 18.1 months<br>2. 12.0 months<br><br>Intracranial objective response rate (ORR-IC):<br>1. 47%<br>2. 20%<br><br>Tucatinib reduced the risk of intracranial progression or death by 68% and the risk of death by 42%.

Supplement Figure S2. Voting results (Agree, Agree with edits, Disagree, Abstain)

1.1	For patients with HER2+ early breast cancer cT1a and b (i.e., ≤1 cm) without evidence of nodal disease (cN0), the standard of care is timely surgery followed by adjuvant treatment depending on the pathologic staging of disease (see 1.3, 1.4, 1.5). [Strong recommendation]	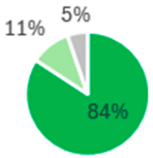
1.2	For patients with HER2+ early breast cancer cT1c (i.e., >1 to ≤2 cm) without evidence of nodal disease (cN0), the standard of care is surgery followed by adjuvant treatment [Strong recommendation]; however, due to current global practices, consideration can be given to neoadjuvant treatment followed by surgery and adjuvant treatment. [Strong consideration]	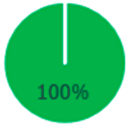
1.3	"For patients with HER2+ early breast cancer with pT1 without evidence of nodal disease (pN0), the standard of care adjuvant systemic treatment is paclitaxel + trastuzumab for 12 weeks followed by trastuzumab monotherapy for 9 months (APT regimen). [Strong recommendation]<br><br>Other non-anthracycline-based chemotherapy regimens (i.e., docetaxel + carboplatin or docetaxel + cyclophosphamide) + trastuzumab can be considered. [Strong recommendation]"	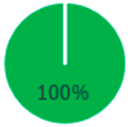
1.4	Although neoadjuvant treatment is preferred, for those patients who are treated with upfront surgery and are then found to have ≥pT2 pN0 disease, the standard of care is adjuvant chemotherapy + trastuzumab. [Strong recommendation]	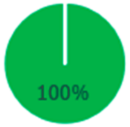
1.5	Although neoadjuvant treatment is preferred, for those patients who are treated with upfront surgery and are then found to have nodal disease in the pathological specimen (pN+), the standard of care is adjuvant chemotherapy + trastuzumab with consideration given to pertuzumab. [Strong consideration]	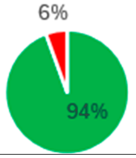
1.6	Although there is not a survival benefit, for patients with HER2+ HR+ and N+ disease who have completed (neo)adjuvant chemotherapy + trastuzumab, extended adjuvant treatment with neratinib for 1 year after completion of trastuzumab-based adjuvant therapy can be considered to decrease recurrence. [Moderate recommendation]	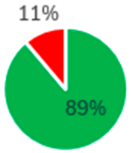

1.7	For patients with HER2+ early breast cancer with ≥cT2 or those with nodal disease (cN+), the standard of care is neoadjuvant therapy with chemotherapy + trastuzumab + pertuzumab. [Strong recommendation]	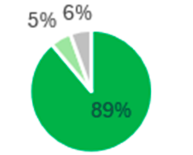
1.8	For patients with HER2+ early breast cancer in whom a pathological complete response is determined in the surgical specimen after completion of neoadjuvant chemotherapy + trastuzumab + pertuzumab, the standard of care is trastuzumab for a total of 1 year. [Strong recommendation]	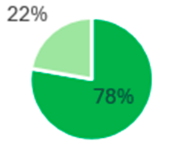
1.9	For patients with HER2+ early breast cancer in whom residual invasive disease is detected pathologically in the surgical specimen of the breast or axillary lymph nodes after completion of neoadjuvant chemotherapy + trastuzumab + pertuzumab, the standard of care is to treat with trastuzumab emtansine (T-DM1) for 14 cycles in the adjuvant setting. [Strong recommendation]	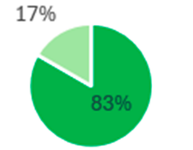
2.1	When safe and feasible, repeat biopsy should be performed in all patients whose disease relapses on or after adjuvant treatment. [Strong consideration]	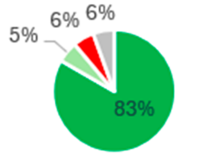
2.2	For patients with de novo HER2+ (HR±) metastatic breast cancer who have not received prior HER2-directed therapy or chemotherapy for metastatic disease the standard of care is trastuzumab + pertuzumab + taxane chemotherapy for 6-8 cycles followed by trastuzumab + pertuzumab maintenance therapy. [Strong recommendation]	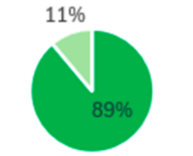
2.3	For patients with HER2-positive (HR±) metastatic breast cancer whose disease relapses >6 mos after completion of (neo)adjuvant chemotherapy + HER2-directed therapy, the standard of care is trastuzumab + pertuzumab + taxane chemotherapy for 6-8 cycles followed by trastuzumab + pertuzumab maintenance therapy. [Strong recommendation]	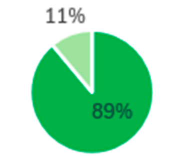
2.4	For patients with HER2-positive (HR±) metastatic breast cancer whose disease relapses on or ≤6 months after completion of (neo)adjuvant chemotherapy + HER2-directed therapy, the recommended treatment is as per the second-line recommendation. [Strong recommendation]	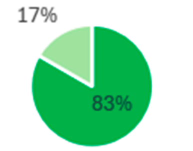

2.5	For patients with HER2-positive metastatic breast cancer whose disease has progressed on first-line HER2-directed therapy, the standard of care is trastuzumab deruxtecan (T-DXd) in the absence of contraindications. [Strong recommendation]	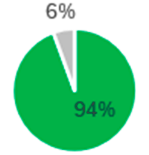
2.6	For patients with HER2+ metastatic breast cancer whose disease has progressed after at least two HER2-directed therapies the recommendation for treatment is tucatinib + capecitabine + trastuzumab (and can be considered earlier if brain metastasis is present). [Moderate recommendation]	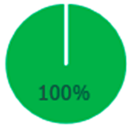
2.7	For patients with HER2+ metastatic breast cancer whose disease has progressed after at least three HER2-directed therapies, the recommendation based on evidence is to continue HER2-directed therapy. Options include: [Moderate recommendation]	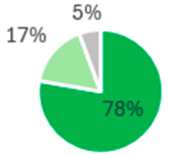
3.1	For patients with HER2+ metastatic breast cancer, screening for CNS metastasis(es) should be considered in asymptomatic patients at baseline in the metastatic setting and at disease progression [Strong consideration]; In symptomatic patients, appropriate screening investigations for CNS metastasis(es) are essential. [Strong recommendation]	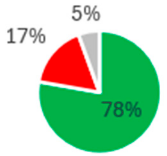
3.2	For patients with a history of HER2+ metastatic breast cancer who are diagnosed with brain metastasis(es), multidisciplinary care with representation from radiology, radiation oncology, neurosurgery, medical oncology, and supportive care is the standard of care with the multidisciplinary team deciding on sequencing of local and systemic therapies. [REAL Alliance expert opinion]	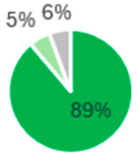
3.3	"For patients with a history of HER+ metastatic breast cancer but without evidence of systemic disease (i.e., NED) who present with oligometastatic brain disease amenable to local therapy, there is insufficient evidence to make a recommendation for systemic therapy. Multidisciplinary care* is the standard of care, and the multidisciplinary team is to decide on sequencing of local and systemic therapies in such patients. [REAL Alliance expert opinion]<br><br>* Includes radiologist (neuroradiologist, if available), radiation oncologist, neurosurgeon, medical oncologist, supportive care"	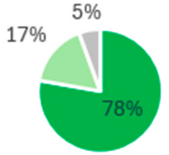

3.4	For patients with a history of HER2+ metastatic disease who present with asymptomatic, low volume, newly diagnosed brain metastasis(es), treatment should be discussed by MDT incorporating patient values with treatment options including initial HER2-directed systemic therapy versus upfront local therapy. [REAL Alliance expert opinion]	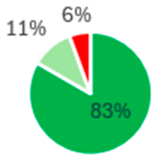
3.5	For patients with a history of HER2+ metastatic disease who present with symptomatic, newly diagnosed brain metastasis(es), upfront stereotactic radiosurgery (SRS) is a reasonable approach (preferred over whole brain radiotherapy) [REAL Alliance expert opinion]	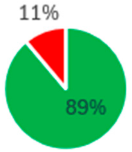
3.6	"For patients with HER2+ metastatic breast cancer with parenchymal CNS disease, the decision to offer systemic therapy prior to local therapies should be individualized for each patient and ideally discussed at multidisciplinary* rounds. Key considerations include tumour burden and clinical symptoms. A multidisciplinary approach should be conducted to confirm if and when systemic therapy should be held during local CNS therapy to reduce the risk of toxicities and tumour necrosis. [REAL Alliance expert opinion]<br><br>* Includes radiologist (neuroradiologist, if available), radiation oncologist, neurosurgeon, medical oncologist, and supportive care."	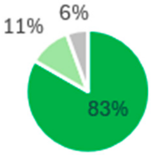
3.7	For patients with HER+ metastatic breast cancer with active or progressive systemic disease in the presence of treated brain metastasis(es), the standard of care in the first line setting is trastuzumab + pertuzumab + taxane. [Strong recommendation]	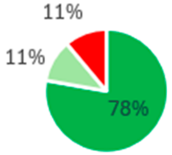
3.8	For patients with HER2+ metastatic breast cancer with stable brain metastasis(es) whose disease has progressed on first line therapy, the standard of care options are T-DXd (preferred) or tucatinib + capecitabine + trastuzumab. [Strong recommendation]	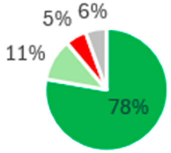
3.9	For patients with HER2+ metastatic breast cancer and asymptomatic active (i.e, untreated) or stable brain metastasis(es) where local therapy is not indicated whose disease has progressed on first-line therapy, options include tucatinib + capecitabine + trastuzumab or T-DXd. Such cases should be reviewed by the multidisciplinary team to determine sequencing of local and systemic therapies. [Strong recommendation]	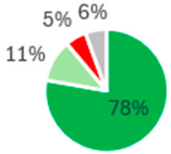

3.10	"For patients with HER2-positive metastatic breast cancer and active brain metastasis(es) whose disease has progressed on second-line systemic therapy, the standard of care is as follows: [Strong recommendation]<br><br>Tier 1 regimens:<br>Tucatinib + capecitabine + trastuzumab (preferred if not used in second line)<br>T-DXd (preferred if not used in second line or if there are signs of extensive systemic disease)<br>Tier 2 regimens: only if local intervention is not feasible<br>T-DM1<br>Neratinib + capecitabine"	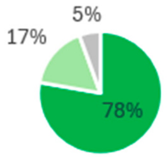
4.1	In patients with HER2+ metastatic breast cancer, a drug holiday can be considered in cases where there is stable disease on imaging. [REAL Alliance expert opinion]	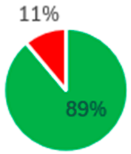
4.2	In patients with HER2+ de novo metastatic breast cancer who have a clinical CR in the metastatic sites from chemotherapy + HER2-directed therapy and whose PET scan is negative, but who have breast-only residual disease, local therapy (surgery and/or RT) can be considered at the site of the primary tumour in order to achieve NED. [REAL Alliance expert opinion]	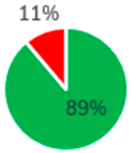
